# Tektin bundle interacting protein, TEKTIP1, functions to stabilize the tektin bundle and axoneme in mouse sperm flagella

**DOI:** 10.1007/s00018-023-05081-3

**Published:** 2024-03-07

**Authors:** Xin-Yan Geng, Hui-Juan Jin, Lan Xia, Bin-Bin Wang, Su-Ren Chen

**Affiliations:** 1 Key Laboratory of Cell Proliferation and Regulation Biology, Ministry of Education, Department of Biology, College of Life Sciences, Beijing Normal University, Beijing, 100875 People’s Republic of China; 2 Center for Genetics, National Research Institute of Family Planning, Beijing, 100081 China; 3 Graduate School of Peking Union Medical College &, Chinese Academy of Medical Sciences, Beijing, 100005 China; 4 NHC Key Laboratory of Reproductive Health Engineering Technology Research (NRIFP), National Research Institute for Family Planning, Beijing, 100081 China

**Keywords:** Sperm motility, Microtubule inner protein, Tektins, TEKTIP1, Knockout mice

## Abstract

Tektins are microtubule inner proteins (MIPs) and localize at the inside lumen of doublet microtubules (DMTs) of cilia/flagella. TEKTIP1, a newly identified protein by cryo-electron microscopy (cryo-EM), is proposed to be localized at the center of the tektin bundle and hypothesized to recruit tektins or stabilize the bundle. However, the physiological role of TEKTIP1 is unknown. In this study, we generated *Tektip1*-knockout (*Tektip1*^−/−^) mice and showed that they were male subfertile primarily due to reduced sperm motility. A high percentage of sperm from *Tektip1*^−/−^ mice showed moderately disorganized axoneme structures and abnormal flagellar waveforms. TEKTIP1 predominately interacted with TEKT3 among tektins. Loss of TEKTIP1 partially disturbed the organization of tektin bundle by mainly affecting the native status of TEKT3 and its interaction with other tektins. Collectively, our study reveals the physiological role and potential molecular mechanism of TEKTIP1 in axonemal structure and sperm motility, highlights the importance of MIPs in stabilizing DMTs, and suggests a potential relevance of TEKTIP1 deficiency to human asthenospermia. *Tektip1*^−/−^ mice will be an excellent animal model to study the DMT organization of sperm flagella using cryo-EM in future.

**Supplementary Information:**

The online version contains supplementary material available at 10.1007/s00018-023-05081-3.

## Introduction

Cilia and flagella are evolutionarily conserved organelles that play central roles in motility and other biological events in diverse cell types and organs [1]. Dysfunction of cilia and flagella is highly associated with a variety of human diseases, including primary ciliary dyskinesia (PCD) and multiple morphological abnormalities of the sperm flagella (MMAF). PCD (MIM: 244400) refers to a multisystem disorder that includes chronic airway diseases, situs inversus, hydrocephalus, and/or sterility. MMAF (MIM: 617576), a subtype of astheno-teratozoospermia, is characterized by short, coiled, absent, and/or irregular flagella without any other symptoms of PCD.

The axoneme, the core of motile cilia and flagella, consists of a well-organized ‘9 + 2’ microtubule structure with nine DMTs surrounding a central pair singlet microtubule [2]. MIPs are localized at the luminal surfaces of DMTs [3, 4]. Given that MIPs bind to the inside of microtubules, they are proposed to assemble and/or stabilize DMTs [5]. Cryo-EM studies have identified many MIPs in mammalian cilia and flagella [3, 4]. Despite genetic studies in mice showing that ablation of MNS1 [6] or CFAP53 [7], two MIPs, causes loss of outer dynein arms from axonemes and disrupts ciliary motility, the physiological significance of other MIPs is largely unclear.

Tektins are insoluble α-helical proteins and are among the most abundant and conserved ciliary/flagella proteins [8]. Tektins form stable polymers that are proposed to resemble intermediate filaments [9]; however, cryo-EM study considers tektins (helix-turn-helix organization) and intermediate filaments (coiled-coil architecture) to be structurally distinct classes of cytoskeletal filaments [4]. A pentagonal tektin bundle formed by TEKT1/2/3/4 filaments is revealed by the cryo-EM structural analysis of bovine respiratory DMTs [4]. Structural comparison between mammalian sperm DMT and respiratory DMTs reveals that nearly all MIPs in respiratory DMTs are present in the sperm DMT, including the pentagonal tektin bundle formed by TEKT1/2/3/4 filaments [10, 11]. A unique feature of mouse sperm DMTs is the presence of seven classes of TEKT5 based on their positions and orientations [10, 11]. Tektin organizations also slightly differ between species. For example, TEKT3 homodimer that forms the pentagonal arrangement of tektin filaments within the A tubule of bovine DMTs is missing in humans [12]. Only four classes of TEKT5 are present in the human sperm DMTs, which have fewer MIPs compared with the mouse sperm DMTs [10, 11].

To reach and fuse with the egg, sperm must complete a long journey through the female reproductive tract powered by its flagella [13]. The pathogenic mutations in genes coding for axonemal proteins are well-known genetic factors accounting for male infertility with asthenospermia [14, 15]. Tektins have been reported to be critical for sperm motility and male fertility using knockout mouse models. *Tekt2*-knockout mice exhibit male infertility due to impaired inner arm dynein function [16]. The absence of TEKT4 causes subfertility with drastically reduced motility but grossly unaltered flagellar ultrastructure in mice [17]. Although *Tekt3*-knockout mice exhibit normal male fertility, they produce sperm with reduced motility and increased flagellar structural beading defects [18]. A recent study identified two homozygous *TEKT3* mutations in infertile men with oligo-astheno-teratozoospermia [19], highlighting the relevance of tektins deficiency to male infertility.

A cryo-EM study published in *Cell* in 2021 [4] identified TEKTIP1, a functionally unknown protein, is proposed to be localized at the center of the tektin bundle. Given its central position, TEKTIP1 is hypothesized to function to recruit tektins or stabilize the bundle. However, the physiological roles of TEKTIP1 are unknown due to a lack of animal evidence. Does TEKTIP1 play essential roles in the tektin bundle, axoneme structure and sperm motility? To address this question, we generated *Tektip1*-knockout mice and found that they were male subfertile due to a disruption in sperm motility. The waveform of sperm flagella and ultrastructure of axonemes were moderately altered after the loss of TEKTIP1. The impact of TEKTIP1 deficiency on the expression and organization of tektins was further investigated at molecular levels.

## Results

### TEKTIP1 is enriched in the testis and distributed in sperm flagella

**Fig. 1:**
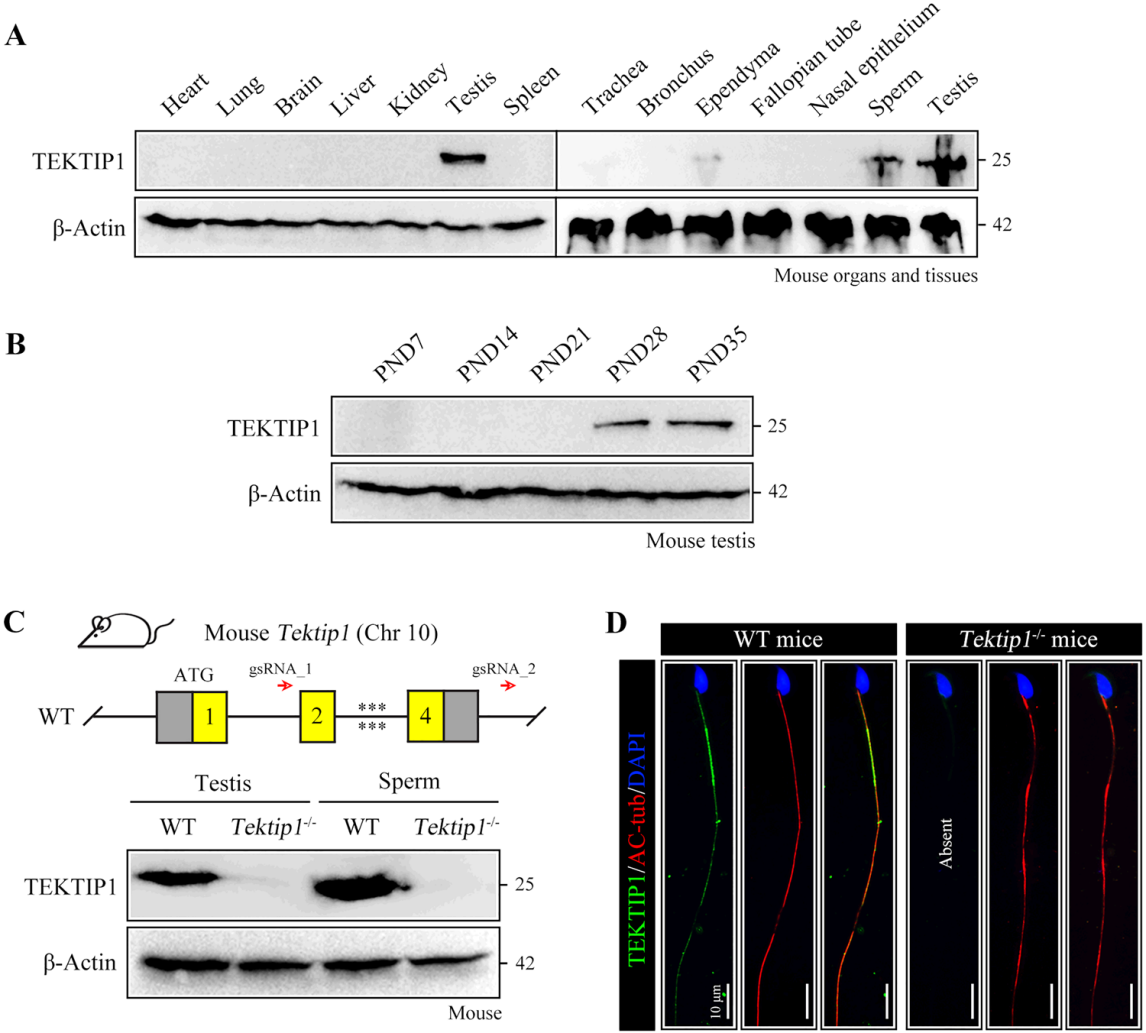
Expression of TEKTIP1 and establishment of *Tektip1*^−/−^ mice. **A** Mouse TEKTIP1 expression in specified organs/tissues, detected via Western blotting and normalized to β-Actin. **B** Mouse TEKTIP1 expression in postnatal testes detected via Western blotting and normalized to β-Actin. PND, postnatal day. **C** Genomic features and knockout strategy of mouse *Tektip1*. Detailed information on the construction of *Tektip1*^−/−^ mice is provided in Supplementary Fig. S3. TEKTIP1 protein expression in testis and sperm samples from *Tektip1*^−/−^ mice and WT mice. β-Actin served as an internal control. **D** Immunofluorescence staining of sperm from WT mice and *Tektip1*^−/−^ mice using anti-TEKTIP1 (green) and anti-AC-tub (acetylated-tubulin, red) antibodies. The nucleus was stained with DAPI (blue). Scale bar, 10 μm

Using sequence alignment analysis, we demonstrated that TEKTIP1 was conserved among mammals, including humans, chimpanzees, dogs, cattle, mice, rats, horses, and domestic ferrets (Supplementary Fig. 1). According to the Human Protein Atlas, human *TEKTIP1* mRNA was highly expressed in the testis (Supplementary Fig. 2A). By single-cell type analysis, *TEKTIP1* mRNA was found to be restricted to early and late spermatids within the human testis (Supplementary Fig. 2B). Using TEKTIP1 antiserum generated by our laboratory, we showed that TEKTIP1 was localized at full-length flagella of human sperm (Supplementary Fig. 2C). According to mouse ENCODE transcriptome data, *Tektip1* mRNA was predominantly expressed in the testes (Supplementary Fig. 2D). In mice, TEKTIP1 was abundant in the testis and sperm, and a very low level of TEKTIP1 was detected in trachea and ependyma; undetectable levels of TEKTIP1 were revealed in other organs/tissues, including the heart, lung, whole brain, liver, kidney, spleen, bronchus, fallopian tube, and nasal epithelium (Fig. 1A). We further revealed that TEKTIP1 is expressed in mouse testes starting from approximately postnatal day 28 (Fig. 1B), which corresponded with the first detection of haploid spermatids. These expression analyses indicate that TEKTIP1 may function in cilia/flagella development and/or function.

### Generation of *Tektip1*^−/−^ mice

Given the proposed central position of TEKTIP1 in the tektin bundle, TEKTIP1 is hypothesized to recruit tektins or stabilize the bundle [4]. To elucidate its physiological function, we established a *Tektip1*^−/−^ mouse line, using the CRISPR‒Cas9 genome editing system (Fig. 1C). In short, two sgRNAs that targeted exons 2 ~ 4 were administered to zygotes in combination with Cas9 mRNA. The founder mice were then mated with wild-type (WT) females, thereby giving rise to heterozygous mice carrying a 2141-bp deletion in the *Tektip1* gene (Supplementary Fig. 3). *Tektip1*^−/−^ mice were obtained by mating heterozygous mice and subsequent genotyping. TEKTIP1 expression was absent in the protein lysates of testes and sperm from *Tektip1*^−/−^ mice (Fig. 1C). TEKTIP1 was localized along the sperm flagella of WT mice, while its expression disappeared in the sperm flagella of *Tektip1*^−/−^ mice (Fig. 1D).

### *Tektip1*^−/−^ male mice are subfertile

**Fig. 2:**
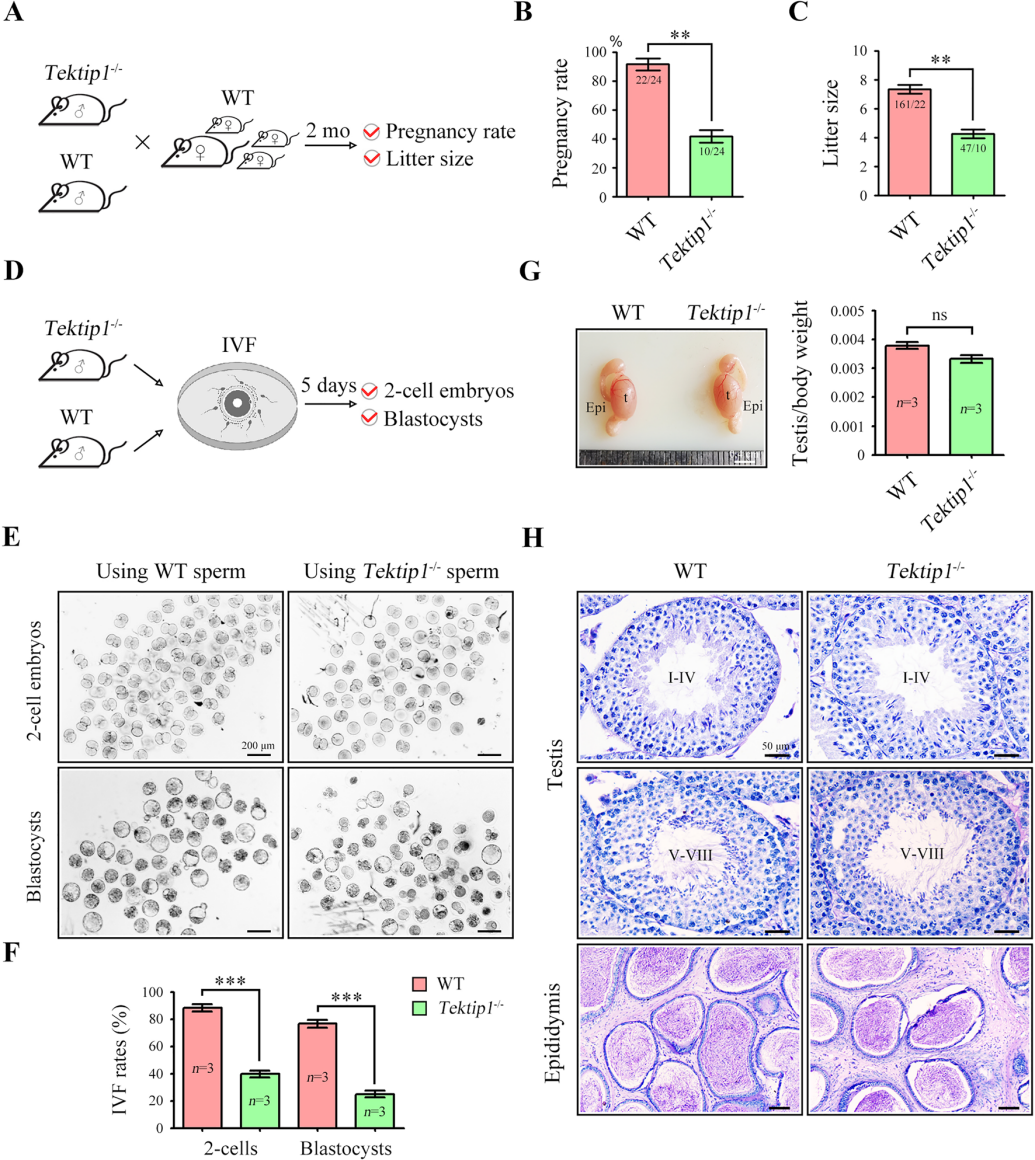
Fertilizing ability and sperm parameters of *Tektip1*^−/−^ mice. **A** Adult *Tektip1*^−/−^ male mice and their littermate WT mice (*n* = 3 each group) were continuously coupled with adult WT female mice at a sex ratio of 1:2 for 2 months. **B** The pregnancy rate was calculated. Data are represented as the mean ± SD, Student’s *t* test (unpaired, two-tailed), **p < 0.01. **C** The litter size was calculated. Data are represented as the mean ± SD, Student’s *t* test (unpaired, two-tailed), **p < 0.01. **D** An in vitro fertilization (IVF) assay was performed using sperm from adult *Tektip1*^−/−^ mice and WT mice (*n* = 3 for each group). **E** The images of two-cell embryos and blastocyst. **F** The percentage of two-cell embryos and blastocysts was calculated. Data are represented as the mean ± SD, Student’s *t* test (unpaired, two-tailed), ***p < 0.001. **G** Representative images of the testis and epididymis. Evaluation of the weight ratio of the testes/body from adult *Tektip1*^−/−^ mice and WT mice (*n* = 3 for each group). *ns* not significant, *t* testis, *Epi* epididymis. Scale bar, 0.4 cm. **H** Histological evaluation of the testis and epididymis from adult *Tektip1*^−/−^ and WT mice. Scale bar, 50 μm

*Tektip1*^−/−^ male mice were healthy and developed with no identifiable abnormalities, including laterality abnormalities, hydrocephalus, or respiratory problems. Normal development in cilia and axoneme of ependyma and trachea from *Tektip1*^−/−^ mice was revealed by scanning electron microscopy (SEM) and transmission electron microscopy (TEM) (Supplementary Fig. 4). To examine the fertility of male mice, we mated *Tektip1*^−/−^ male mice and their littermate WT male mice with WT female mice for 2 months (Fig. 2A). Although *Tektip1*^−/−^ male mice were able to mate normally with females, both the pregnancy rate of mating females and pups that were born from *Tektip1*^−/−^ males were significantly reduced (Fig. 2B, C). We next performed in vitro fertilization (IVF) using sperm of *Tektip1*^−/−^ mice or their littermate WT mice (Fig. 2D). The percentages of two-cell embryos and blastocysts using sperm of *Tektip1*^−/−^ mice were also significantly decreased (Fig. 2E, F). Accordingly, *Tektip1*^−/−^ male mice are subfertile. We further dissected the male reproductive system and performed the histological examination. The testis size and weight were similar between *Tektip1*^−/−^ mice and their littermate WT mice (Fig. 2G). Histological examination of testis and cauda epididymis from *Tektip1*^−/−^ mice further revealed a generally normal spermatogenesis and sperm production (Fig. 2H).

### TEKTIP1 deficiency disturbs axonemal ultrastructure, flagellar waveform, and sperm motility

**Fig. 3:**
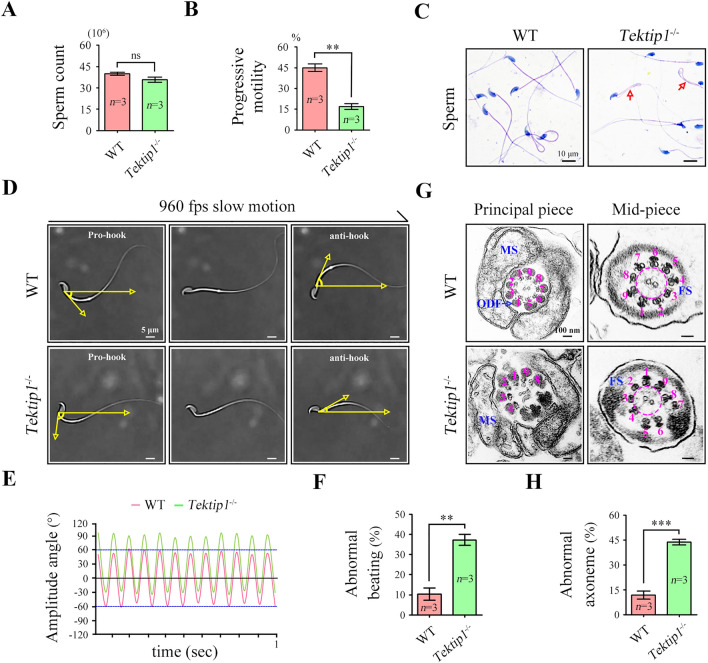
Flagellar waveform and ultrastructural assessment of sperm from *Tektip1*^−/−^ mice. **A** Sperm counts were determined using a fertility counting chamber under a light microscope. Data are represented as the mean ± SD (*n* = 3 each group), Student’s *t* test (unpaired, two-tailed), ns, not significant. **B** Progressive motility was assessed via computer-assisted semen analysis (CASA). Data are represented as the mean ± SD (*n* = 3 each group), Student’s *t* test (unpaired, two-tailed), **p < 0.01. **C** Light microscopy analysis of sperm from *Tektip1*^−/−^ mice and WT mice using Papanicolaou staining. Red arrows indicated bent-tail sperm. Scale bar, 10 μm. **D** Flagellar waveforms of head-tethered sperm from *Tektip1*^−/−^ mice and WT mice. Scale bar, 5 μm. **E** The amplitude angle of flagellar waveforms was recorded for 1 s. Flagellar waveforms of sperm from WT mice were symmetrical (± 60°). Flagellar waveforms of sperm from *Tektip1*^−/−^ mice were asymmetrical (-30° ~ 90°). **F** Percentage of sperm showing abnormal beating patterns from *Tektip1*^−/−^ mice and WT mice. One hundred spermatids in each group were counted. Data are represented as the mean ± SD (*n* = 3 each group), Student’s *t* test (unpaired, two-tailed), **p < 0.01. **G** Transmission electron microscope (TEM) analysis showing the principle piece and mid-piece of sperm flagella from *Tektip1*^−/−^ mice and WT mice. ODF, outer dense fibre; MS, mitochondrial sheath; FS, fibrous sheath. Scale bar, 100 nm. **H** Percentage of sperm showing abnormal axoneme structures from *Tektip1*^−/−^ mice and WT mice. Twenty-five spermatids in each group were counted. Data are represented as the mean ± SD (*n* = 3 each group), Student’s *t* test (unpaired, two-tailed), ***p < 0.001

We next examined semen characteristics and sperm morphology. Although the sperm count was similar between *Tektip1*^−/−^ mice and WT mice (Fig. 3A), the progressive motility of sperm was markedly reduced in *Tektip1*^−/−^ mice (~ 16%) as compared with WT mice (~ 45%) (Fig. 3B). The morphology of sperm from *Tektip1*^−/−^ mice was generally normal; however, bent tails were frequently observed (arrows indicated in Fig. 3C). We further characterized the flagellar beat of single sperm by tethering their heads to the glass surface. A symmetric flagellar beat was observed in sperm of WT mice, whereas the flagella of some sperm from *Tektip1*^−/−^ mice were beating asymmetrically (Fig. 3D). The amplitude angle of sperm of WT mice was between 60° and -60°, while it was between 90° and -30° in sperm of *Tektip1*^−/−^ mice (Fig. 3E). The percentage of sperm showing abnormal beating was significantly higher in *Tektip1*^−/−^ mice (~ 38%) than in WT mice (~ 10%) (Fig. 3F). Moreover, we observed the ultrastructure of axoneme by TEM. Compared with the well-organized “9 + 2” axoneme in sperm of WT mice, some DMTs in the principle piece and mid-piece of sperm flagella from *Tektip1*^−/−^ mice were dislocated or partially missing (Fig. 3G). The percentage of sperm exhibiting abnormal axoneme ultrastructure in *Tektip1*^−/−^ mice (~ 42%) was significantly greater than that in WT mice (~ 12%) (Fig. 3H). Collectively, these data suggest that *Tektip1*^−/−^ male mice are subfertile mainly due to a higher percentage of sperm exhibiting disordered axonemal ultrastructure and abnormal sperm motility.

### Proteomic analysis of sperm from *Tektip1*^−/−^ mice and WT mice

To try to determine the molecular basis of the deficiency of sperm motility after the loss of TEKTIP1, we first applied quantitative iTRAQ (isobaric tags for relative and absolute quantification) and mass spectrometry to identify the differentially expressed (DE) proteins of sperm from adult *Tektip1*^−/−^ mice and WT mice (*n* = 3 samples each group) (Supplementary Fig. 5A). A cut-point of 1.5-fold change and a *p* value (student’s *t* test) less than 0.05 were selected. Compared with WT group, TEKTIP1 deletion only caused a significant decrease of 32 and a significant increase of 28 proteins in sperm (Supplementary Fig. 5B). All DE proteins were listed in Supplementary Table S1 and the mass spectrometry proteomics data have been deposited to ProteomeXchange with the dataset identifier PXD044492. Among significantly downregulated sperm proteins in *Tektip1*^−/−^ mice, the enriched pathways primarily included ‘seminal vesicle proteins/sperm capacitation’, ‘male fertility/cilia & flagella’, ‘ribosomal proteins’, and ‘extracellular matrix’, as revealed by Kyoto Encyclopedia of Genes and Genomes (KEGG) and presented as protein–protein interaction networks using STRING (Supplementary Fig. 5C). Gene set enrichment analysis (GSEA) showed significant enrichment (|Normal ES|> 1, Normal *p* value < 0.05, FDR < 0.25) in ‘spermatogenesis’, ‘cilium/flagella movement’, and ‘sperm capacitation/fertilization’ among all DE sperm proteins between WT mice and *Tektip1*^−/−^ mice (Supplementary Fig. 5D). Given that there is no evidence to indicate the direct regulation of these DE proteins by TEKTIP1, we suggest that the alternation of protein expression may be secondary effects. Moreover, the protein levels of tektins and identified MIPs in mouse sperm lysates did not differ between *Tektip1*^−/−^ mice and WT mice (Supplementary Table S2), indicating that loss of TEKTIP1 may affect the organization rather than the expression of tektins and MIPs.

### TEKTIP1 interacts with TEKT3 but not TEKT1, TEKT2, or TEKT4

**Fig. 4:**
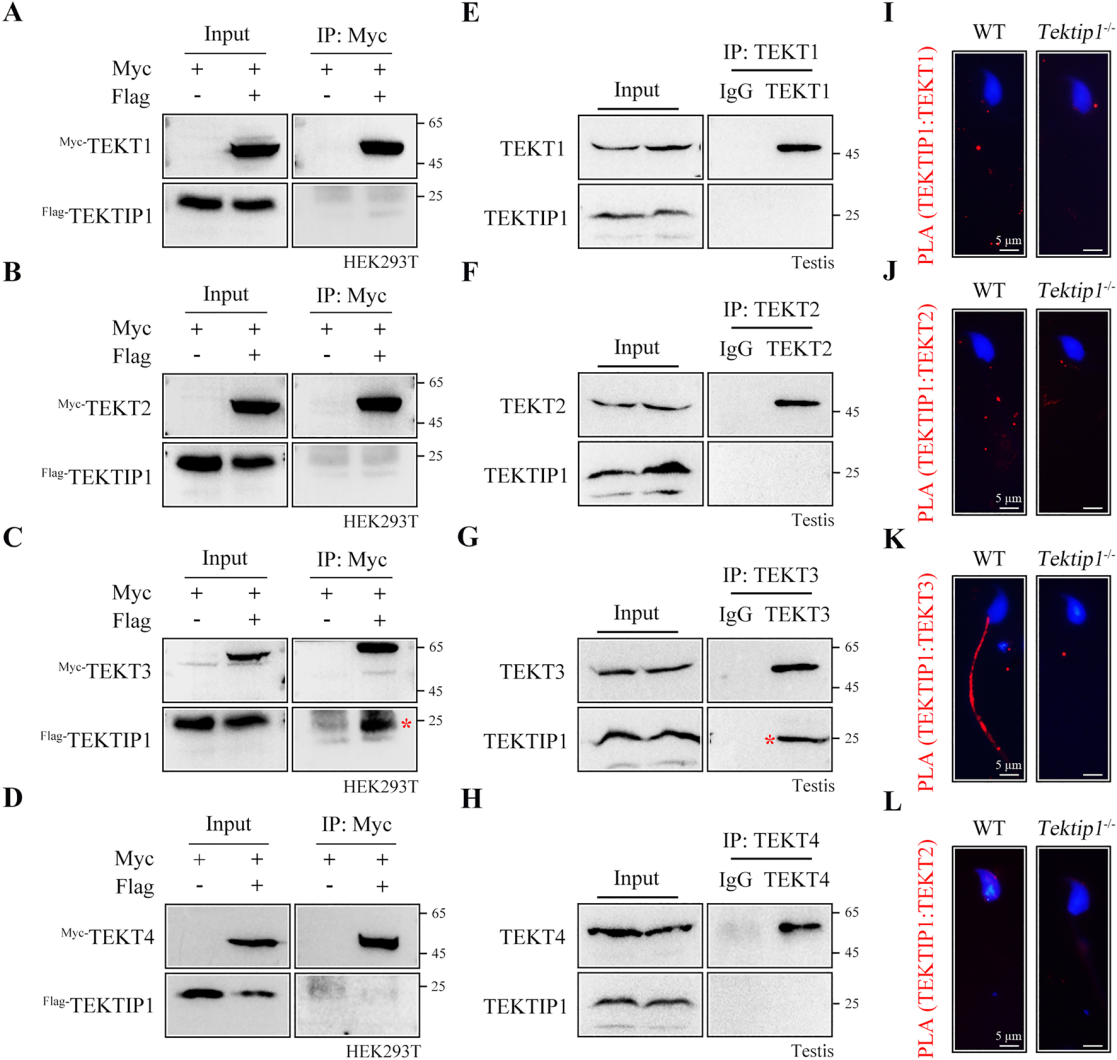
TEKTIP1 interacts with TEKT3 but not TEKT1, TEKT2, or TEKT4. **A–D** Myc-tagged plasmids of TEKT1 ~ 4 and Flag-tagged plasmid of TEKTIP1 were constructed. Flag-tagged TEKTIP1 was immunoprecipitated with Myc-tagged TEKT3 but not Myc-tagged TEKT1, TEKT2, or TEKT4 in HEK293T cell lysates using co-IP assays. **E–H** An interaction between endogenous TEKTIP1 and TEKT3 was identified in mouse testis lysates using a co-IP assay. Endogenous TEKTIP1 was not immunoprecipitated with TEKT1, TEKT2, or TEKT4. Rabbit IgG served as the negative control. **I-L** Representative immunofluorescence images from a PLA performed on sperm from WT and *Tektip1*.^−/−^ mice. Closely interaction was revealed by positive signals (red). Sperm heads were stained with DAPI (blue)

Although TEKTIP1 is proposed to be located at the center of the tektin bundle, as revealed by cryo-EM [4], the interaction between TEKTIP1 and tektins is still unclear. By coimmunoprecipitation (co-IP) assays, we showed that Flag-tagged TEKTIP1 was immunoprecipitated with Myc-tagged TEKT3 in the protein lysates of HEK293T cells (Fig. 4C). However, no detectable interactions between Flag-tagged TEKTIP1 and Myc-tagged TEKT1, TEKT2, or TEKT4 could be identified (Fig. 4A, B and D). The TEKTIP1-TEKT3 connection was further confirmed by endogenous co-IP using mouse testis extracts (Fig. 4G). Similarly, TEKTIP1 could not be immunoprecipitated by TEKT1, TEKT2, or TEKT4 in mouse testis lysates (Fig. 4E, F and H). We investigated this further using a proximity ligation assay (PLA) [20]. Using TEKTIP1 and TEKT3 antibodies, PLA revealed a positive staining along the sperm flagella of WT mice but not *Tektip1*^−/−^ mice (Fig. 4K). Closely interaction between TEKTIP1 and TEKT1/2/4 in sperm flagella was also not revealed by PLA (Fig. 4I, J, L). The tight interaction of TEKTIP1 with TEKT3 but not other tektins indicates that loss of TEKTIP1 may influence the tektin bundle organization primarily through TEKT3.

### Loss of TEKTIP1 interferes TEKT3 organization and its connection with other tektins

**Fig. 5:**
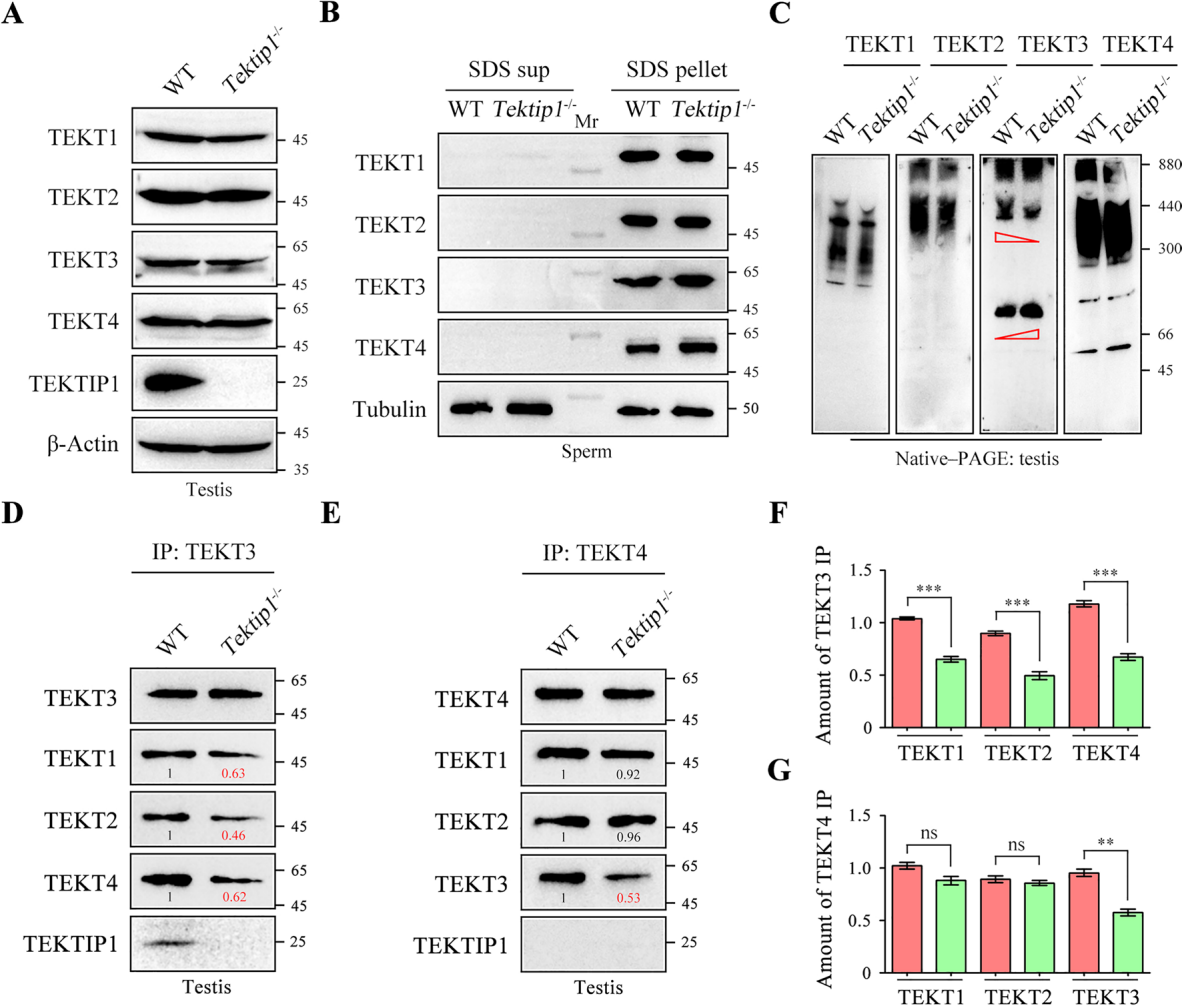
Expression and connections of tektins after the loss of TEKTIP1. **A** Expression of TEKT1 ~ 4 was analysed by Western blotting (SDS‒PAGE) in testis lysates of adult *Tektip1*^−/−^ mice and WT mice. β-Actin served as a loading control. **B** Sperm samples were treated with SDS-EDTA solution. Supernatant and pellet samples were resolved by SDS‒PAGE. Expression of TEKT1 ~ 4 was analysed by Western blotting (SDS‒PAGE) in sperm lysates of adult *Tektip1*^−/−^ mice and WT mice. **C** Expression of TEKT1 ~ 4 was analysed by Western blotting (Native‒PAGE) in testis lysates of adult *Tektip1*^−/−^ mice and WT mice under native conditions. **D** Endogenous TEKT3 was immunoprecipitated from testis protein lysates of *Tektip1*^−/−^ mice and WT mice. The amounts of TEKT1, TEKT2, or TEKT4 coimmunoprecipitated by TEKT3 were further detected by Western blotting. **E** The amounts of TEKT1, TEKT2, or TEKT3 coimmunoprecipitated by endogenous TEKT4 were detected in testis protein lysates of *Tektip1*^−/−^ mice and WT mice. **F** Bar graph representing the band intensities of TEKT3-immunoprecipitated TEKT1, TEKT2, or TEKT4. Data represent the mean ± SD (*n* = 3 each group), Student’s *t* test (unpaired, two-tailed), ^***^p < 0.001. **G** Bar graph representing the band intensities of TEKT4-immunoprecipitated TEKT1, TEKT2, or TEKT3. Data represent the mean ± SD (*n* = 3 each group), Student’s *t* test (unpaired, two-tailed), ^**^p < 0.01; ns, not significant

Sperm proteomic analysis indicated that loss of TEKTIP1 did not alter the protein levels of tektins (Supplementary Table S2). Western blotting further confirmed that the protein levels of TEKT1 ~ 4 in mouse testis lysates did not obviously changed between WT mice and *Tektip1*^−/−^ mice (Fig. 5A). After treatment of sperm samples with SDS-EDTA solution, TEKT1 ~ 4 were predominantly distributed in the pellet (Fig. 5B). Both the constituent distributions and expression of TEKT1 ~ 4 in sperm were similar between *Tektip1*^−/−^ mice and WT mice (Fig. 5B).

Although loss of TEKTIP1 does not affect the expression of tektins in samples of testis and sperm, TEKTIP1 may regulate the organization of tektin bundles. The expression patterns of tektins were further examined using native‒PAGE. Compared with those in WT mice, the content level of TEKT3 high polymers was reduced, while its oligomer expression was increased in testis protein lysates of *Tektip1*^−/−^ mice (Fig. 5C). Furthermore, the polymers of TEKT1, 2, 4 were also slightly less abundant in testis protein lysates of *Tektip1*^−/−^ mice (Fig. 5C). We further examined the interaction between TEKT3 and other tektins in testis protein lysates of WT mice and *Tektip1*^−/−^ mice by co-IP assays. The amounts of TEKT3-immunoprecipitated TEKT1, TEKT2, or TEKT4 were significantly reduced in *Tektip1*^−/−^ mice (Fig. 5D and F). In contrast, the amounts of TEKT4-immunoprecipitated TEKT1 or TEKT2 were indistinguishable between WT mice and *Tektip1*^−/−^ mice (Fig. 5E and G). Collectively, these data suggest that loss of TEKTIP1 predominantly affects the native organization of TEKT3 and its connections with other tektins, further influencing the integrity and stability of tektin bundles.

## Discussion

MIPs are localized at the luminal surfaces of nine DMTs, which are core axonemal components of cilia and flagella. Emerging cryo-EM studies provide valuable information for the presence of MIPs and their spatial organization [4, 10–12, 21–23]. It is speculated that MIPs function to assemble and/or stabilize the tubulin-organized axoneme; however, their physiological roles are largely unclear.

Tektin bundles are well-known MIPs inside DMTs and mainly contain tektin 1–4. A recent cryo-EM study discovered that TEKTIP1, a functionally unknown protein, is proposed to be localized at the center of the tektin bundle [4]. TEKTIP1 is thus hypothesized to function to recruit tektins or stabilize the bundle; however, animal evidence is lacking to explore its physiological role in the motility of cilia/flagella.

Our current study showed that *Tektip1*^−/−^ mice were subfertile primarily due to impaired sperm motility. The ultrastructure of the axoneme was moderately disorganized in the sperm flagella of *Tektip1*^−/−^ mice. *Tektip1*^−/−^ mice were not totally infertile, indicating that deletion of a single TEKTIP1 may not severely affect the stability of the axoneme. Similarly, *Tekt4*-knockout also causes male subfertility with reduced motility but grossly unaltered flagellar ultrastructure in mice [17]. *Tekt3*^−/−^ mice produce sperm with reduced motility and increased flagellar structural beading defects but not male infertility [18]. *Tekt5*^−/−^ mice are still fertile but have a lower fraction of motile sperm and a higher percentage of defective sperm flagella with 180° bends [24].

Although TEKTIP1 was first identified in bovine respiratory cilia by cryo-EM [4], it is predominantly expressed in the human and mouse testis, as indicated by public database and our Western blotting analysis. Within the testis, TEKTIP1 is restricted to early/late spermatids and specifically localized along the sperm flagella. *Tektip1*^−/−^ mice do not display obvious symptoms of ciliopathy, including laterality abnormalities, hydrocephalus, and defects of respiratory cilia. We suggest that TEKTIP1 may be selectively required for mouse sperm flagella. The differential requirements of proteins for cilia and flagella are also observed in other knockout mice. For example, IQUB expression is detected in tissues other than the testis, such as the lung and brain; however, IQUB deletion only affects beating of sperm flagella but not respiratory cilia [25].

Our study reveals the potential mechanism of TEKTIP1 in the organization/stability of tektin bundles at molecular level. Although TEKTIP1 is proposed to be localized at the center of the tektin bundle, we found that it only strongly interacts with TEKT3 but not TEKT1, TEKT2, or TEKT4. As co-IP assay could not detect low-affinity interaction, we could not fully exclude the possibility that TEKTIP1 may also interact with other tektins. TEKTIP1 is critical for the organization of TEKT3 because the content level of TEKT3 polymers was reduced while its monomer expression was increased in testis protein lysates of *Tektip1*^−/−^ mice. TEKTIP1 further assists the connection of TEKT3 with other tektins. Attenuated TEKT3-TEKT1, TEKT3-TEKT2, and TEKT3-TEKT4 were observed in the testis protein lysates of *Tektip1*^−/−^ mice. In contrast, the amounts of TEKT4-immunoprecipitated TEKT1 and TEKT2 in the testis protein lysates were similar between *Tektip1*^−/−^ mice and WT mice.

A very recent study [24] analyzes the sperm doublet structure of *Tekt5*^−/−^ mice and compares it with the WT counterpart, providing a first example to apply cryo-EM technology to a knockout mouse strain. Tektin homolog substitute on the same position of 3-helix bundle in the absence of TEKT5; however, the occupancy of broken and curved helical bundles is largely lost [24]. It should be emphasized that a cryo-EM analysis of sperm DMTs from *Tektip1*^−/−^ mice will provide direct evidence to explore the effects of TEKTIP1 on DMT stability. Molecular and biochemical experiments in our current study indirectly indicate that tektin bundle is partially impaired after TEKTIP1 loss. The dislocated or partially missing DMTs identified by TEM analysis of sperm in *Tektip1*^−/−^ mice are most likely to be a secondary effect. It will be of great interest to find out how lack of TEKTIP1 directly affects tektin bundle, its luminal localization, and organization of the adjacent MIPs by applying cryo-EM technology in future.

Zhou et al. recently discovered variants in 10 distinct MIPs (FAM166A, CCDC105, TEX37, EFCAB6, EFHC2, FAM166C, MNS1, TEKT1, CFAP45, and CFAP21) associated with a subtype of asthenozoospermia termed MIP-variant-associated asthenozoospermia (MIVA), which is characterized by impaired sperm motility without evident morphological abnormalities [11]. Furthermore, two homozygous *TEKT3* mutations have been recently identified in infertile men with asthenospermia [19], highlighting the relevance of tektins deficiency to human male infertility. Our animal study suggests that TEKTIP1 is essential for mouse sperm motility and that its deficiency may be a potential genetic cause of human male infertility with MIVA. However, clinical evidence linking TEKTIP1 to male infertility is still lacking. Whole-exome sequencing of a large cohort of infertile men primarily with asthenospermia will be useful to screen any pathogenic mutations in *TEKTIP1*.

Emerging cryo-EM studies resolve axonemal proteins in native mammalian ciliary/flagellar axoneme, which provide valuable information for functional studies. Surprisingly, some MIPs, such as SPACA9, SAXO1, TEKT3, and TEKT5, were structurally important for axoneme as revealed by cryo-EM study; however, their knockout mice are all fertile [18, 24, 26, 27]. Although cryo-EM studies provide valuable information for the presence of MIPs and their spatial organization, functional studies are essential to confirm the physiological roles of candidate (structurally important) MIPs. Accordingly, we suggest that combined cryo-EM and knockout mouse studies are a comprehensive approach to understand the axonemal architecture and the motility function of cilia/flagella.

**Fig. 6:**
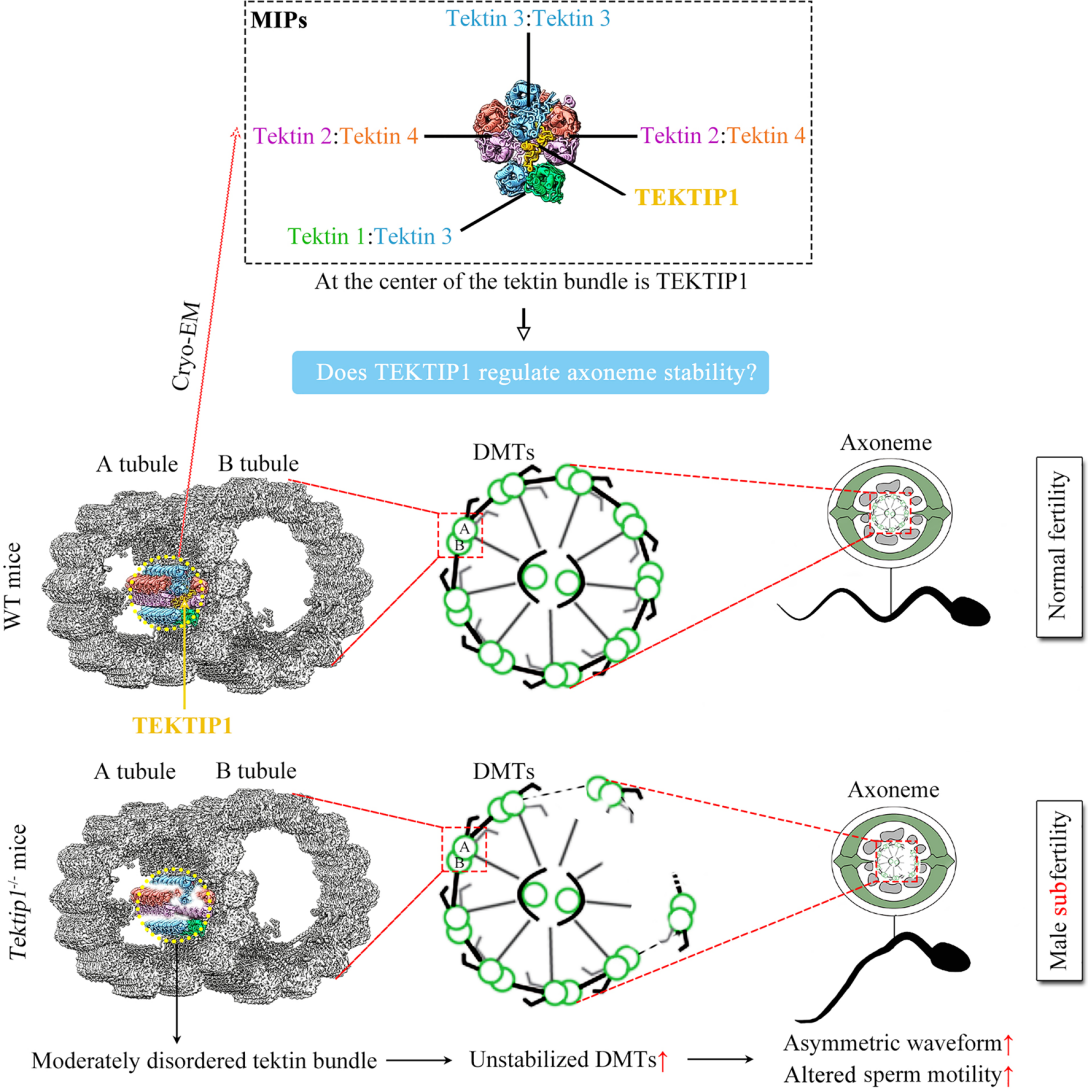
Schematic diagram of the physiological role of TEKTIP1 in tektin bundle, sperm motility and male fertility. Although TEKTIP1 does not regulate the expression of tektins and other MIP proteins, loss of TEKTIP1 causes moderately disordered tektins bundle at the level of protein–protein interaction. As a consequence, the percentage of sperm exhibiting unstabilized DMTs, asymmetric waveform, and altered sperm motility was increased, finally leading to male subfertility of *Tektip1*^−/−^ mice. *MIP* microtubule inner protein, *DMT* doublet microtubule. Some cartoon elements were modified from reference [4, 28].

In summary, our results revealed that TEKTIP1 mainly interacts with TEKT3 within tektin bundles and is critical for its organization and connection with other tektins. Moderate instability of the microtubule inner tektin bundles led to the partially disorganized axoneme of sperm flagella and ultimately altered sperm motility and male subfertility (Fig. 6).

## Methods

### Generation of ***Tektip1***^−/−^ mice

Animal experiments were approved by the Animal Care and Use Committee of the College of Life Sciences, Beijing Normal University. The mouse *Tektip1* gene has 4 transcripts and is located on the chromosome 10. Exons 2 ~ 4 of the *Tektip1-201* (ENSMUST00000020456.5) transcript were selected as the knockout region. Mouse zygotes were coinjected with an RNA mixture of Cas9 mRNA (TriLink BioTechnologies, CA, USA) and sgRNAs. The injected zygotes were transferred into pseudopregnant recipients to obtain the F0 generation. DNA was extracted from tail tissues from 7-day-old offspring and PCR amplification was carried out with genotyping primers using the Mouse Tissue Direct PCR Kit (Tiangen Biotech, China). A stable F1 generation (heterozygous mice) was obtained by mating positive F0 generation mice with wild-type C57BL/6JG-pt mice. The gRNA sequence, genotyping, and Sanger sequencing were provided in Supplementary Fig. 3.

### Expression plasmids and transient transfection

Full-length cDNA encoding TEKTIP1, TEKT1, TEKT2, TEKT3, or TEKT4 was amplified by PCR and cloned into Flag- or Myc-tagged pCMV vectors (Beyotime, Shanghai, China). HEK293T cells (ATCC, NY, USA) were cultured at 37 ℃ in a 5% CO_2_ incubator with Dulbecco’s modified Eagle’s medium with 10% fetal bovine serum and 1% penicillin‒streptomycin (Gibco, NY, USA). Transient transfection of HEK293T cells was performed using Lipofectamine 3000 transfection reagent (Invitrogen, Shanghai, China). Primers for amplification are listed in Supplementary Table 3.

### Generation of TEKTIP1 antibody

Full-length mouse TEKTIP1 was cloned into the pET-N-His-C-His vector (Beyotime, Shanghai, China) and then transfected into the ER2566 E. coli strain (Weidi Biotechnology, Shanghai, China). Protein expression was induced by 1 mM IPTG (Beyotime) at 30 ℃ overnight. After centrifugation, the bacterial pellet was resuspended in buffer (50 mM Tris–HCl pH 8.0, 200 mM NaCl), and the proteins were released by sonication. After centrifugation, anti-His beads (Beyotime) were added to the supernatant and incubated overnight at 4 ℃. After washing, recombinant protein was eluted with 250 mM imidazole (Beyotime). Coomassie brilliant blue stain of the gel of purified TEKTIP1 was shown in Supplementary Fig. 6. Recombinant TEKTIP1 protein was emulsified at a 1:1 ratio (v/v) with Freund’s complete adjuvant (Beyotime) and administered subcutaneously into ICR female mice at multiple points. For the subsequent three immunizations, recombinant TEKTIP1 protein was emulsified with incomplete Freund’s adjuvant (Beyotime) at an interval of 2 weeks. One week after the last immunization, blood was collected, and the serum was separated.

### Fertility testing

To confirm the fertility of *Tektip1*^−/−^ mice, natural mating tests were conducted. Briefly, adult *Tektip1*^−/−^ male mice and their littermate WT mice (*n* = 3 each) were mated with WT C57BL/6 J females (male: female = 1:2) for two months. The vaginal plugs of the mice were examined every morning. Female mice with vaginal plugs were separately fed, and female mice were replenished. The number of pups per litter was recorded.

### Histological analysis

The testes and caudal epididymis were dissected and fixed in 4% PFA overnight at 4 ℃. Fixed tissues were embedded in paraffin, sectioned (5 μm thick), dewaxed, and rehydrated. The sections were stained with Periodic Acid Schiff’s solution (Solarbio, Beijing, China) before imaging using a Leica DM-500 optical microscope (Leica Microsystems, German).

### Sperm count, motility, and morphology

Sperm counts were determined using a fertility counting chamber (Makler, Israel) under a light microscope. Sperm mobility was assessed via the application of a computer-assisted sperm analysis (CASA) system (SAS Medical, China). The sperm suspension was mounted on a glass slide, air-dried, and fixed with 4% PFA for 20 min at room temperature. The slides were stained with Papanicolaou solution (Solarbio, Beijing, China) and observed using a DM500 optical microscope (Leica, Germany).

### Immunofluorescence staining

After permeabilization with 1% Triton X-100 for 1 h, the slides of mouse sperm were blocked with 5% goat serum for 1 h. Mouse TEKTIP1 antiserum (our homemade, 1:100) and rabbit anti-acetylated tubulin (Cell Signaling Technology, #5335, Shanghai, China, 1:100) were added to the slide and incubated overnight at 4 ℃. After washing three times with 1 × PBS, slides were incubated with Alexa Fluor 484-labelled donkey anti-mouse IgG (Beyotime, Shanghai, China, A0428, 1:200) and 555-labelled donkey anti-rabbit IgG (Beyotime, A0453, 1:200) for 1 h at room temperature. The slides were counterstained with DAPI dye and imaged with a fluorescence microscope (Leica Microsystems, Germany).

### Proximity ligation assay (PLA)

Duolink in situ PLAs were carried according to the manufacturer’s instructions (Sigma-Aldrich, DUO920102, CT, USA). Briefly, sperm slides were blocked with Duolink blocking solution for 1 h at 37 ℃. Primary antibodies were diluted in Duolink antibody diluent and incubated overnight at 4 ℃. Mouse TEKTIP1 antiserum (our homemade, 1:100) and rabbit anti-TEKT1 (Proteintech, 18968–1-AP, 1:200), anti-TEKT2 (Proteintech, 13518–1-AP, 1:200), anti-TEKT3 (Proteintech, 12959–1-AP, 1:200), or anti-TEKT4 (Proteintech, 17058–1-AP, 1:200) were used in PLA. Anti-rabbit PLUS and anti-mouse MINUS secondary antibodies were added for 1 h at 37 ℃. A ligation reaction was performed using the Duolink ligation solution and ligase at 37 ℃ for 30 min. Duolink amplification solution and polymerase were utilized for rolling circle amplification and hybridization at 37 ℃ for 1.5 h. Slides were mounted with Duolink mounting medium with DAPI, and imaged with a fluorescence microscope (Leica Microsystems, Germany).

### In vitro fertilization (IVF)

Adult C57BL/6 J female mice were superovulated by injecting 5 IU of pregnant mare serum gonadotropin (PMSG), followed by 5 IU of human chorionic gonadotropin (hCG) 48 h later. Sperm capacitation was performed for 50 min using TYH solution. Cumulus-oocyte complexes (COCs) were obtained from the ampulla of the uterine tube at 14 h after hCG injection. COCs were then incubated with ~ 5 μl sperm suspension (sperm concentration: 1∼5 × 10^6^) in HTF liquid drops at 37 °C under 5% CO_2_. After 6 h, eggs were transferred to liquid drops of KSOM medium. Two-cell embryos and blastocysts were counted at 1 and 4 days postfertilization, respectively. All reagents were purchased from Aibei Biotechnology (Nanjing, China).

### Transmission electron microscopy (TEM)

Mouse sperm were fixed with 2.5% (vol/vol) glutaraldehyde in 0.1 M phosphate buffer (pH 7.4) at 4 ℃. The samples were washed four times in PB and first immersed in 1% (wt/vol) OsO4 and 1.5% (wt/vol) potassium ferricyanide aqueous solution at 4 °C for 2 h. After washing, the samples were dehydrated through graded alcohol into pure acetone. Samples were infiltrated in a graded mixture of acetone and SPI-PON812 resin, and then the pure resin was changed. The specimens were embedded in pure resin with 1.5% BDMA, polymerized for 12 h at 45 °C and 48 h at 60 °C, cut into ultrathin Sects. (70 nm thick), and then stained with uranyl acetate and lead citrate for subsequent observation and photography with a Tecnai G2 Spirit 120 kV (FEI) electron microscope. All reagents were purchased from Zhongjingkeyi Technology (Beijing, China).

### Native‒PAGE

Native‒PAGE gel preparation and electrophoresis were performed using a commercial kit (Real-Timers Biotechnology, Beijing, China). Protein samples were extracted using the Pierce IP Lysis Buffer (Thermo Fisher Scientific, MA, USA), mixed with native gel sample loading buffer (without boiling), and subjected to electrophoresis in Tris–glycine running buffer (without SDS). Thyroglobulin (669 kDa), ferritin (440 kDa), catalase (232 kDa), lactate dehydrogenase (140 kDa), and albumin (66 kDa) were prepared into a native electrophoresis protein marker.

### iTRAQ quantification proteomics

Proteins were extracted from the sperm samples of adult *Tektip1*^−/−^ mice and their littermate WT mice (*n* = 3 each group) using 0.1 M Tris–HCL (pH 8.0), 0.1 M dithiothreitol (DTT), 4% SDS, 1 mM PMSF, and 2% (v/w) protease inhibitor cocktail (Roche, Basel, Switzerland), followed by sonication (20% amplitude, 10 pulses, three times) on ice. The supernatants were collected following centrifugation at 12,000 g for 20 min. Trypsin enzyme solution was added to 100 μg protein samples, vortexed, centrifuged at low speed for 1 min, and incubated at 37 °C for 4 h. The peptide liquid obtained after salt removal was freeze-dried. The peptide sample was dissolved in 0.5 M TEAB and added to the corresponding iTRAQ labelling reagent, followed by storage at room temperature for 2 h. The Shimadzu LC-20AB liquid phase system was used, and the separation column was a 5 μm 4.6 × 250 mm Gemini C18 column for liquid phase separation of the sample. The dried peptide samples were reconstituted with mobile phase A (2% ACN, 0.1% FA) and centrifuged at 20,000 × *g* for 10 min, and the supernatant was taken for injection. Separation was performed by UltiMate 3000 UHPLC (Thermo Fisher). The sample was first enriched in a trap column and desalted, and then entered a self-packed C18 column. The peptides separated by liquid phase chromatography were ionized by a nanoESI source and then passed to a tandem mass spectrometer Q-Exactive HF X (Thermo Fisher) for DDA (Data Dependent Acquisition) mode detection. Raw data were converted to mgf files for bioinformatics analysis, and protein identification from tandem mass spectra was performed by database searching (UniProt). The protein quantification process includes the following steps: protein identification, tag impurity correction, data normalization, missing value imputation, protein ratio calculation, statistical analysis, and results presentation. Proteins with a 1.5-fold change and *p* value (using Student’s *t* test) less than 0.05 were defined as differentially expressed proteins. The mass spectrometry proteomics data have been deposited to the ProteomeXchange Consortium via the iProX partner repository with the dataset identifier PXD044492.

### Western blotting

Proteins from HEK293T cells, mouse tissues, and mouse sperm were extracted using RIPA lysis buffer containing 1 mM PMSF and 2% (v/w) protease inhibitor cocktail (Roche, Basel, Switzerland) on ice. Supernatants were collected following centrifugation at 12,000 × g for 10 min. Proteins were electrophoresed in 10% SDS‒PAGE gels and transferred to nitrocellulose membranes (GE Healthcare, USA). The blots were blocked in 5% milk and incubated with primary antibodies overnight at 4 °C, followed by incubation with secondary antibody for 1 h. For primary antibodies, mouse anti-TEKTIP1 (our homemade, 1:500), rabbit anti-TEKT1 (Proteintech, 18968–1-AP, 1:1000), rabbit anti-TEKT2 (Proteintech, 13518–1-AP, 1:1000), rabbit anti-TEKT3 (Proteintech, 12959–1-AP, 1:1000), or rabbit anti-TEKT4 (Proteintech, 17058–1-AP, 1:1000) were used. Mouse anti-β-Actin (Abcam, ab8226, 1:2000) served as an internal control. For secondary antibodies, goat anti-rabbit IgG H&L (HRP) (Abmart, M212115, 1:5000) or rabbit anti-mouse IgG H&L (HRP) (Abmart, M212131, 1:5000) was utilized. The signals were evaluated using Super ECL Plus Western Blotting Substrate and a Tanon-5200 Multi chemiluminescence imaging system (China).

### SDS-EDTA treatment of sperm

Sperm were homogenized in 1 mL of SDS-EDTA solution (1% SDS, 75 mM NaCl, 24 mM EDTA, pH 6.0) and centrifuged at 5000 × g for 30 min. Then, 100 µL of SDS‒PAGE sample buffer (62.5 mM Tris, pH 6.8, 3% SDS, 10% glycerol, 5% β-mercaptoethanol, 0.02% bromophenol blue) was added to 100 µL of supernatant, while the pellet was resuspended in 200 µL of SDS‒PAGE sample buffer.

### Coimmunoprecipitation (co-IP)

HEK293T cells were transfected with Myc-tagged TEKT1, 2, 3, or 4 alone (control group) or together with Flag-TEKTIP1 (IP group). Forty-eight hours after transfection, cells were lysed with Pierce™ IP Lysis Buffer (Thermo Fisher, CA, USA) containing a 2% (v/w) protease inhibitor cocktail (Roche, Basel, Switzerland) for 30 min at 4 ℃ and then centrifuged at 12,000 × g for 10 min. Protein lysates were incubated overnight with Myc-Tag antibody (Abmart, M20002, 2 μg) at 4 ℃. The lysates were then incubated with 20 μl Pierce™ Protein A/G-conjugated Agarose for 4 h at 4 ℃. The agarose beads were washed five times with Pierce™ IP Lysis Buffer and boiled for 5 min in 1 × SDS loading buffer. Input and IP samples were analysed by Western blotting using HRP conjugated anti-Flag-Tag antibody (Abmart, PA9020, 1:1000) or anti-Myc-Tag antibody (Abmart, M20019, 1:1000). For endogenous co-IP, adult mouse testis tissues were lysed with Pierce™ IP Lysis Buffer. The IP group was treated with rabbit anti-TEKT1 (Proteintech, 18968–1-AP, 1:1000), rabbit anti-TEKT2 (Proteintech, 13518–1-AP, 1:1000), rabbit anti-TEKT3 antibody (Proteintech, 12959–1-AP, 2 μg), or rabbit anti-TEKT4 antibody (Proteintech, 17058–1-AP, 2 μg), and the negative control group was treated with 2 μg rabbit IgG (Beyotime).

### Statistical analyses

Data are presented as the mean ± standard deviation (SD) and were analysed using GraphPad Prism version 5.01 (GraphPad Software). Student’s *t* test (unpaired, two-tailed) was used for the statistical analyses. *p < 0.05, **p < 0.01 and ***p < 0.001.

### Supplementary Information

Below is the link to the electronic supplementary material.

Supplementary file1 (DOCX 4188 KB)

Supplementary file2 (DOCX 6131 KB)

Supplementary file3 (MP4 19420 KB)

Supplementary file4 (MP4 19269 KB)

## Data Availability

The authors declare that all the original data related to the figures and supplementary materials published in this article are available upon rationale request to the corresponding author. All related data are included in either the manuscript or supplementary information.
