# Supplementary figures and images for: Tektin bundle interacting protein, TEKTIP1, functions to stabilize the tektin bundle and axoneme in mouse sperm flagella
Source: Cell Mol Life Sci. 2024 Mar 7;81(1):118. doi: 10.1007/s00018-023-05081-3 (PMC10917850; doi:10.1007/s00018-023-05081-3)

**Original blots**

**
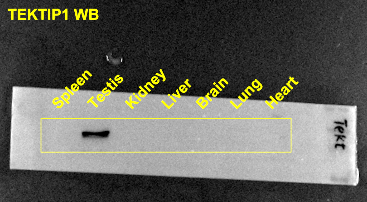

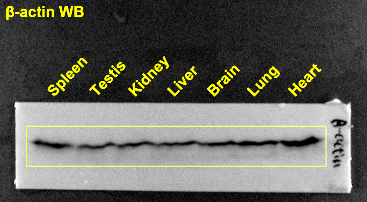
**

**
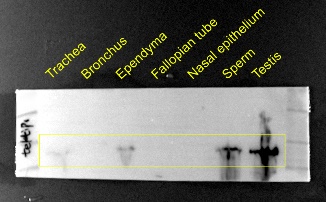

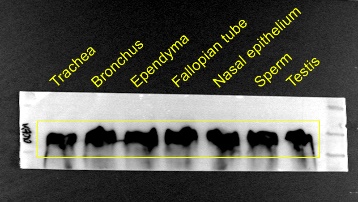
**

**
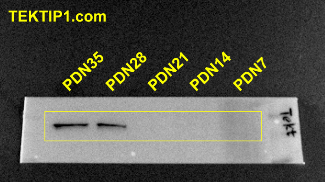

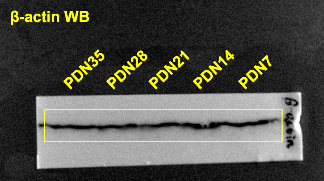

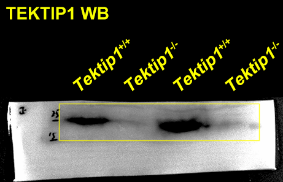

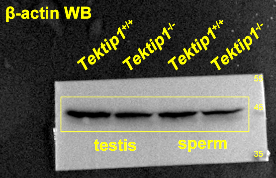
**


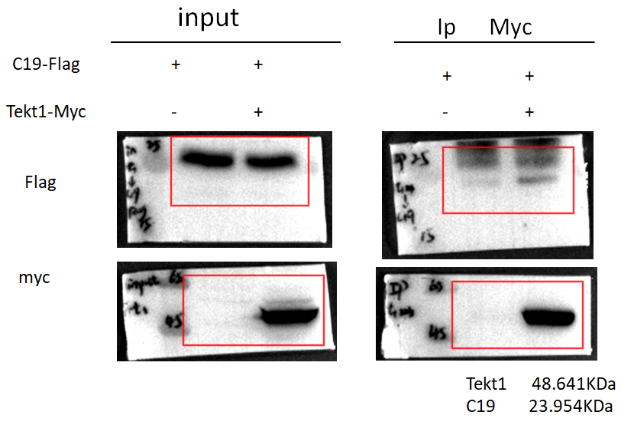

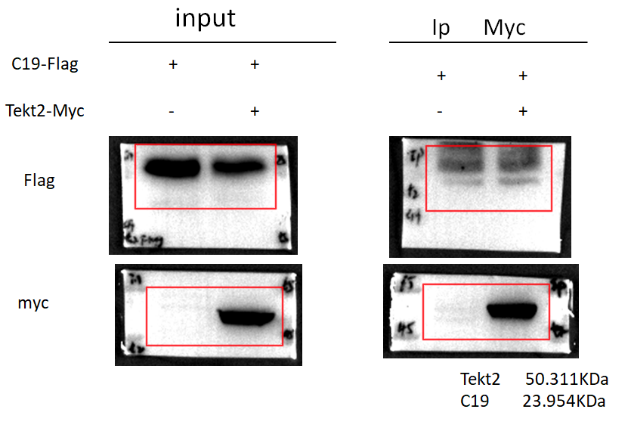


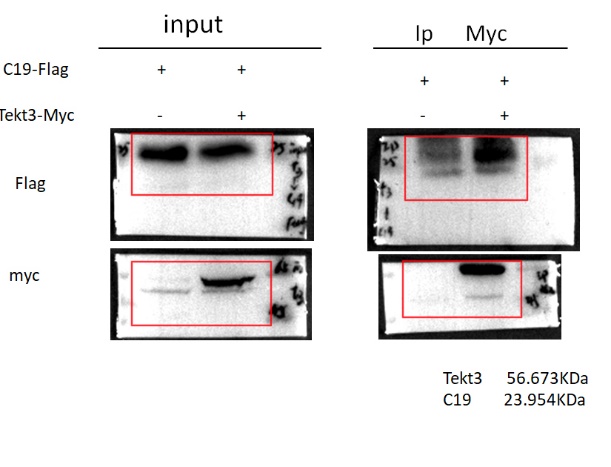

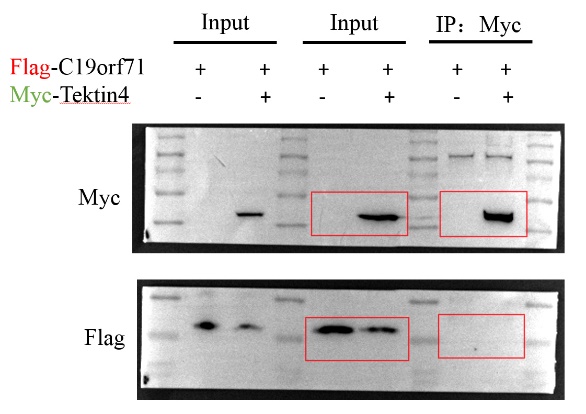


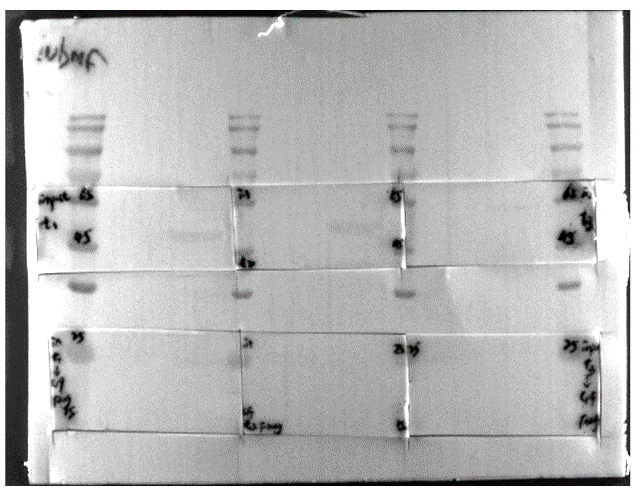

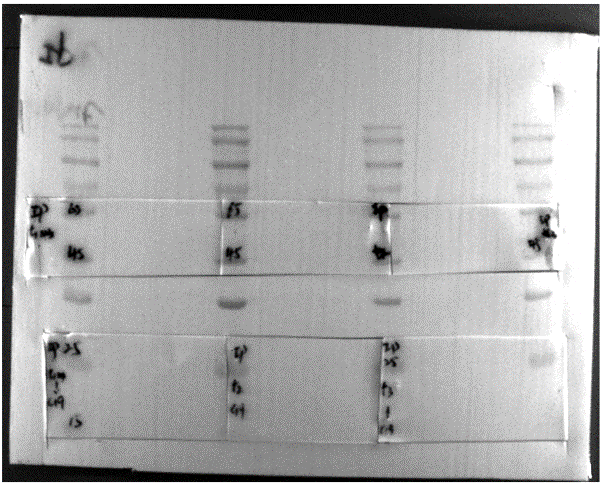





















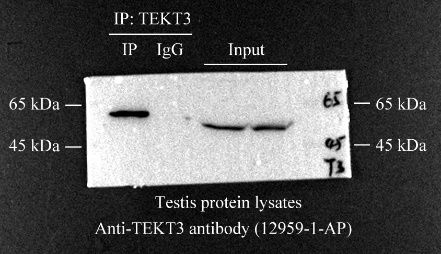

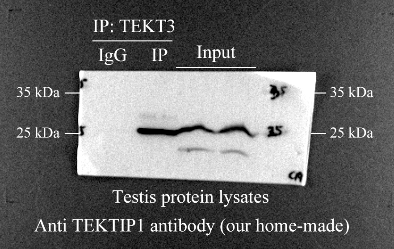











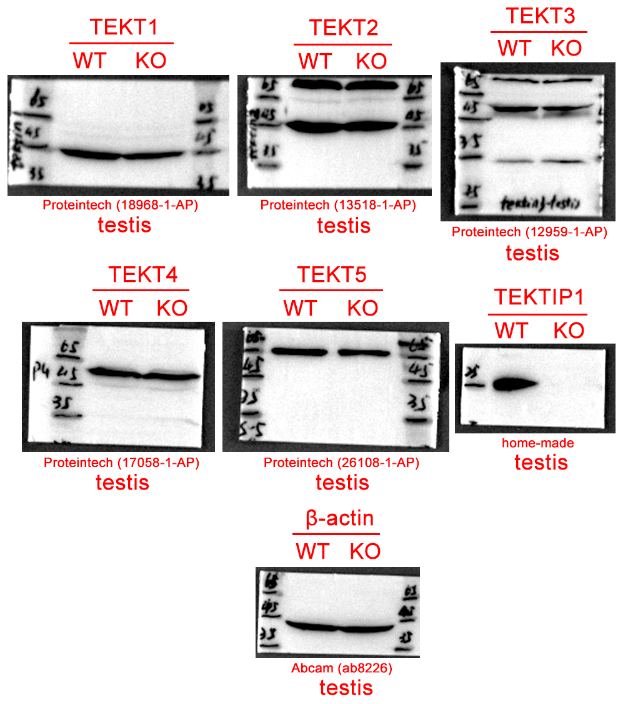


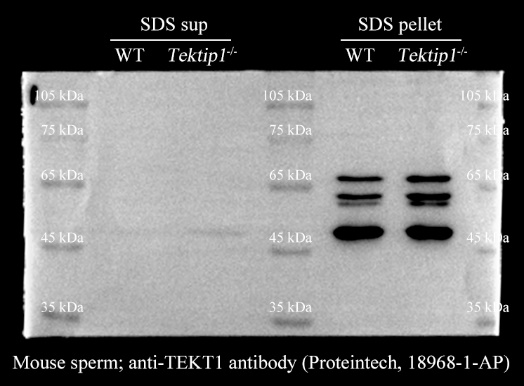

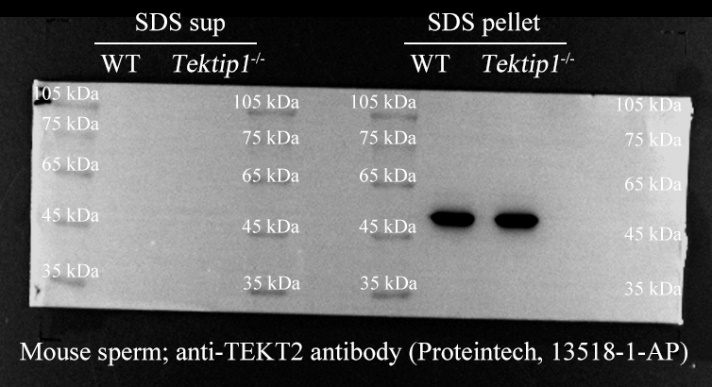


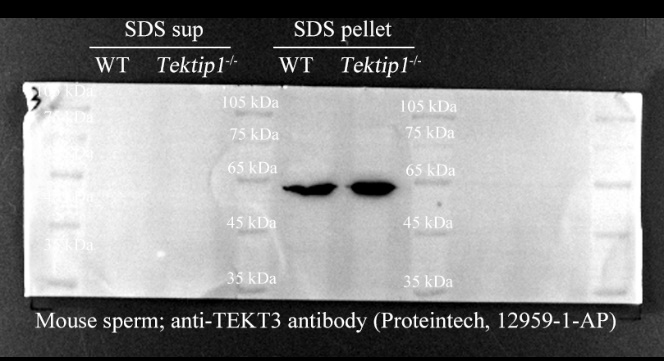

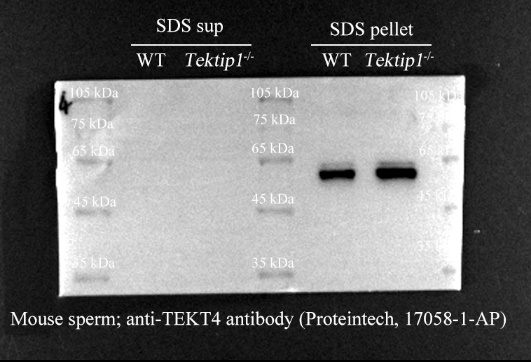


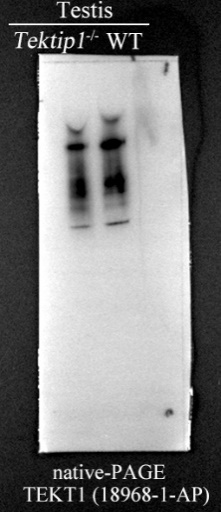

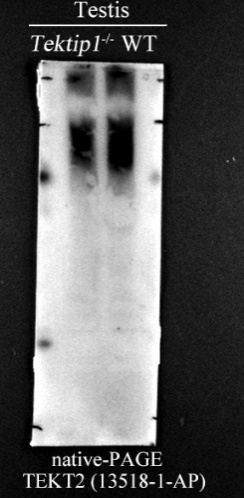

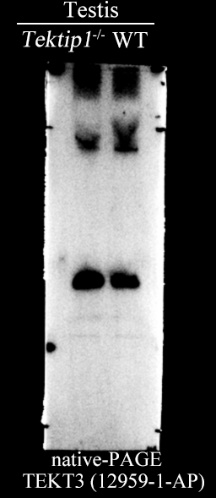

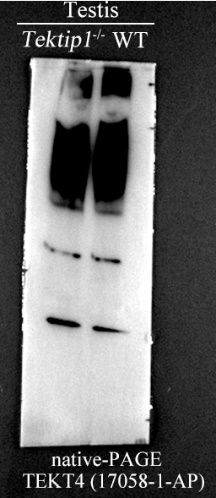


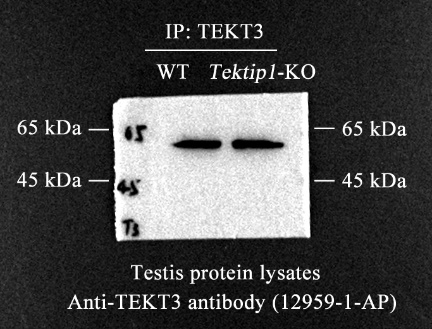




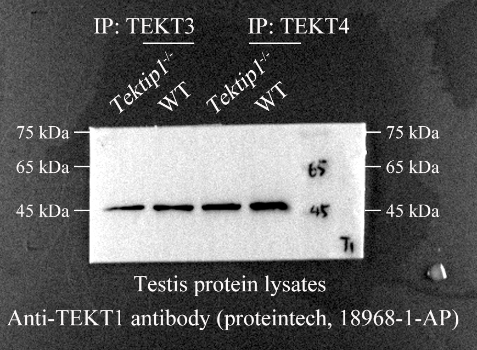

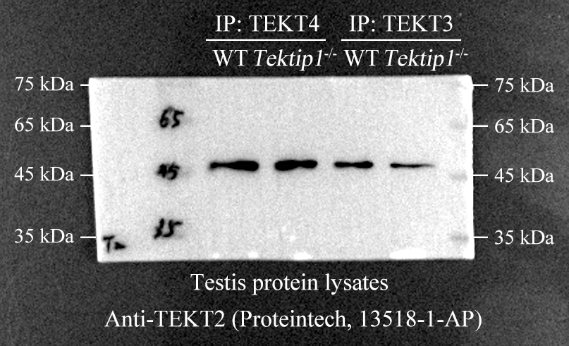


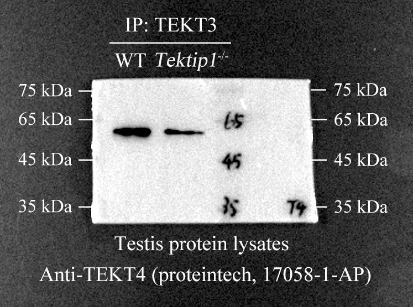




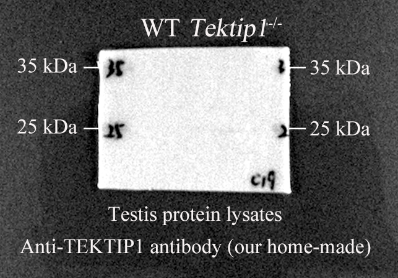

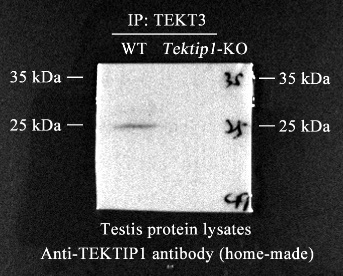

Supplement: Supplementary file 1 — Supplementary file1 (DOCX 4188 KB) [file 18_2023_5081_MOESM1_ESM.docx]
