# Supplementary material for: Tektin bundle interacting protein, TEKTIP1, functions to stabilize the tektin bundle and axoneme in mouse sperm flagella
Source: Cell Mol Life Sci. 2024 Mar 7;81(1):118. doi: 10.1007/s00018-023-05081-3 (PMC10917850; doi:10.1007/s00018-023-05081-3)
Supplement: Supplementary file 2 — Supplementary file2 (DOCX 6131 KB) [file 18_2023_5081_MOESM2_ESM.docx]

**Supplementary Material**

**Tektin bundle interacting protein, TEKTIP1, functions to stabilize the tektin bundle and axoneme in mammalian sperm flagella**

Xin-Yan Geng, Hui-Juan Jin, Lan Xia, Bin-Bin Wang, Su-Ren Chen

The supplementary material contains Figure. S1~S6 and Table S1~S3.


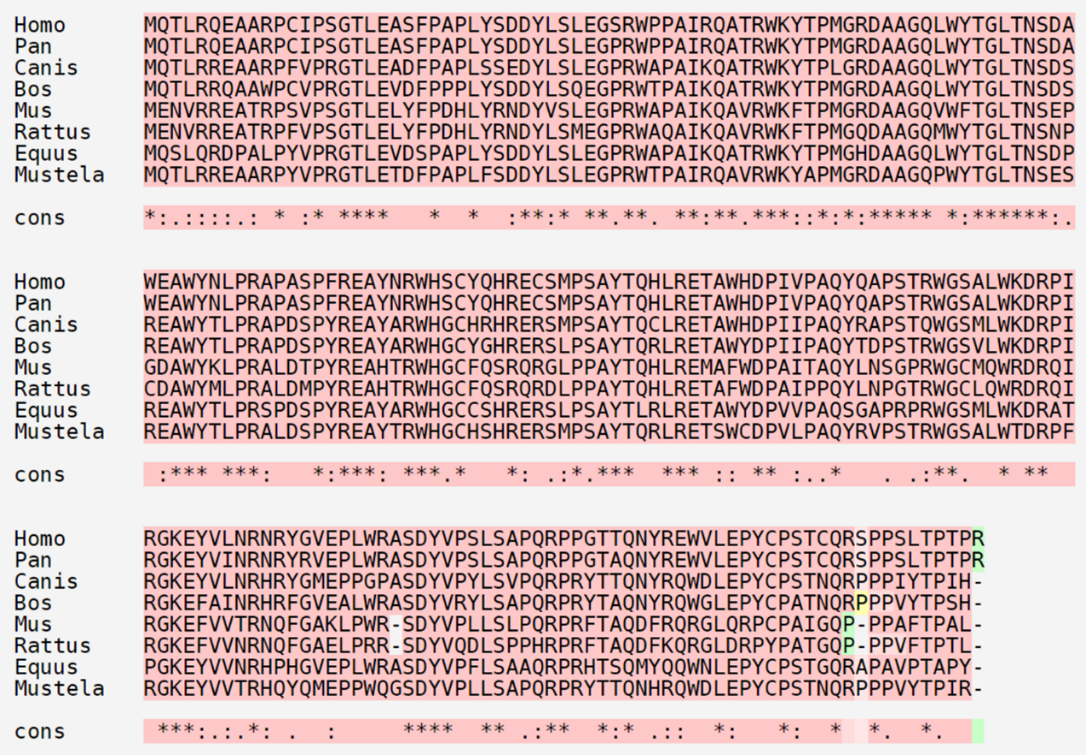


**Fig. S1. TEKTIP1 is conserved among mammals.** Using sequence alignment analysis (M-Coffee, https://tcoffee.crg.eu/apps/tcoffee/do:mcoffee), we revealed that TEKTIP1 was remarkably conserved among human, monkey, dog, cattle, mouse, rat, horse, and weasel.

**
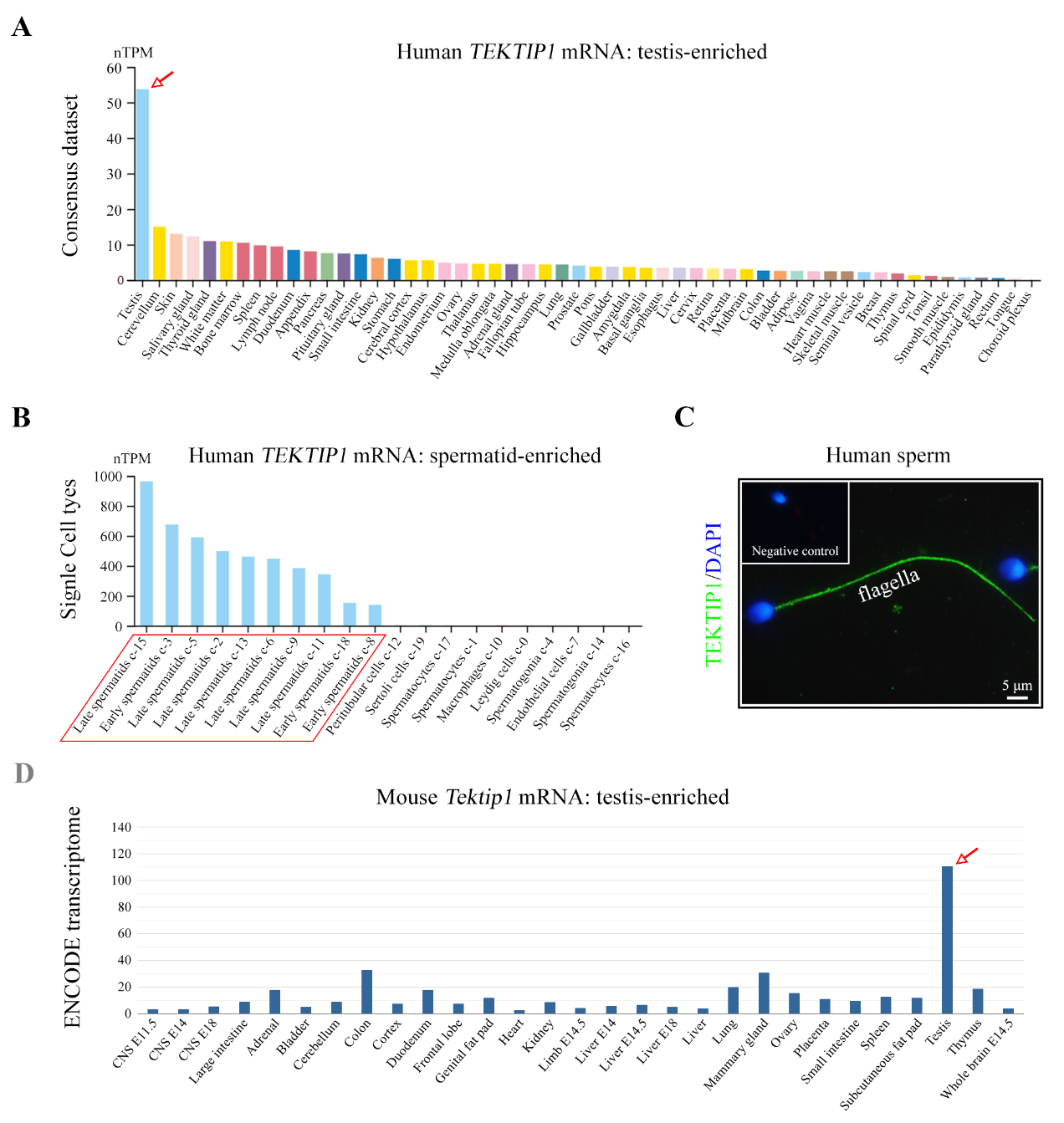
**

**Fig. S2. Expression information of TEKTIP1 in humans and mice.** **A** Among different human tissues, *TEKTIP1* mRNA was predominantly expressed in the testes (https://www.proteinatlas.org/ENSG00000183397-C19orf71/tissue). **B** Human *TEKTIP1* mRNA was restricted to early and late spermatids within the testes (https://www.proteinatlas.org/ENSG00000183397-C19orf71/single+cell+type). **C** Immunofluorescence staining of TEKTIP1 in human sperm samples. For a negative control, IgG was added instead of the primary antibody. The nucleus was stained with DAPI. Scale bar, 5 μm. **D** According to mouse ENCODE transcriptome data (https://www.ncbi.nlm.nih.gov/bioproject/PRJNA66167/), *Tektip1* mRNA was predominantly expressed in the testes.


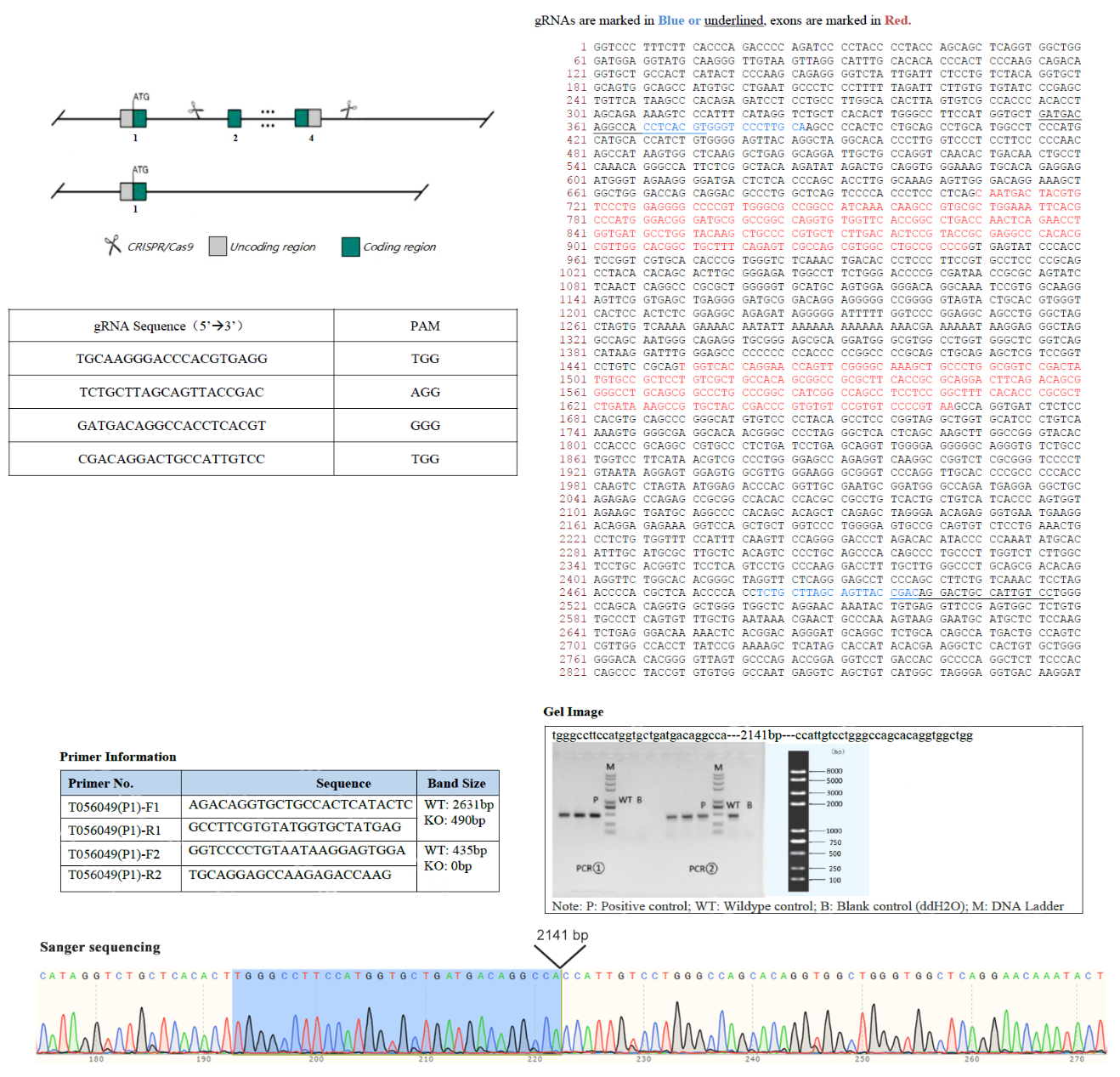


**Fig. S3. Animal report for generation of *Tektip1*^-/-^ mice.** Genomic region of mouse *Tektip1* locus. The mouse *Tektip1* gene (Gene ID: 432479) is located on mouse chromosome 10. Four exons have been identified and exons 2~4 were selected as the knockout region. gRNAs are marked in blue or underlined and exons are marked in red. Cas9 mRNA and gRNAs were co-injected into fertilized eggs for KO mouse production. The pups will be genotyped by PCR followed by sequence analysis. F0 founder animals were identified by PCR and Sanger sequencing. F0 found animals were bred to WT mice to test germline transmission and F1 animal generation. Positive F1 animals were confirmed by PCR and Sanger sequencing. gRNA sequence, primer sequence, gel image, and Sanger sequencing were shown.


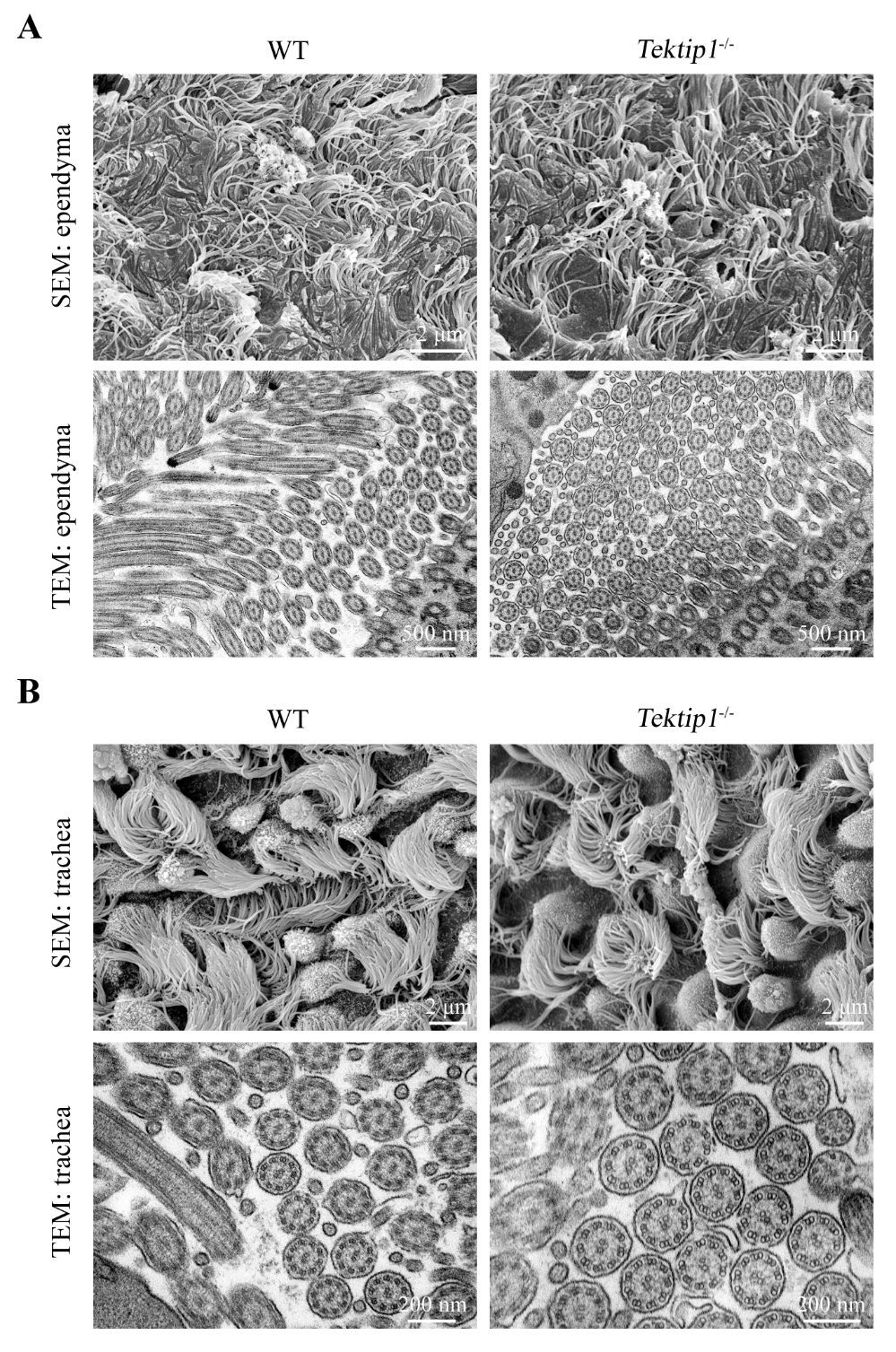


**Fig. S4. Electron microscopy images of cilia from ependyma and trachea. A** Scanning electron microscopy (SEM) and transmission electron microscopy (TEM) of ependymal cilia from WT mice and *Tektip1*^-/-^ mice. Scale bars, 2 μm and 500 nm. **B** Representative SEM and TEM of tracheal cilia from WT mice and *Tektip1*^-/-^ mice. Scale bars, 2 μm and 200 nm.


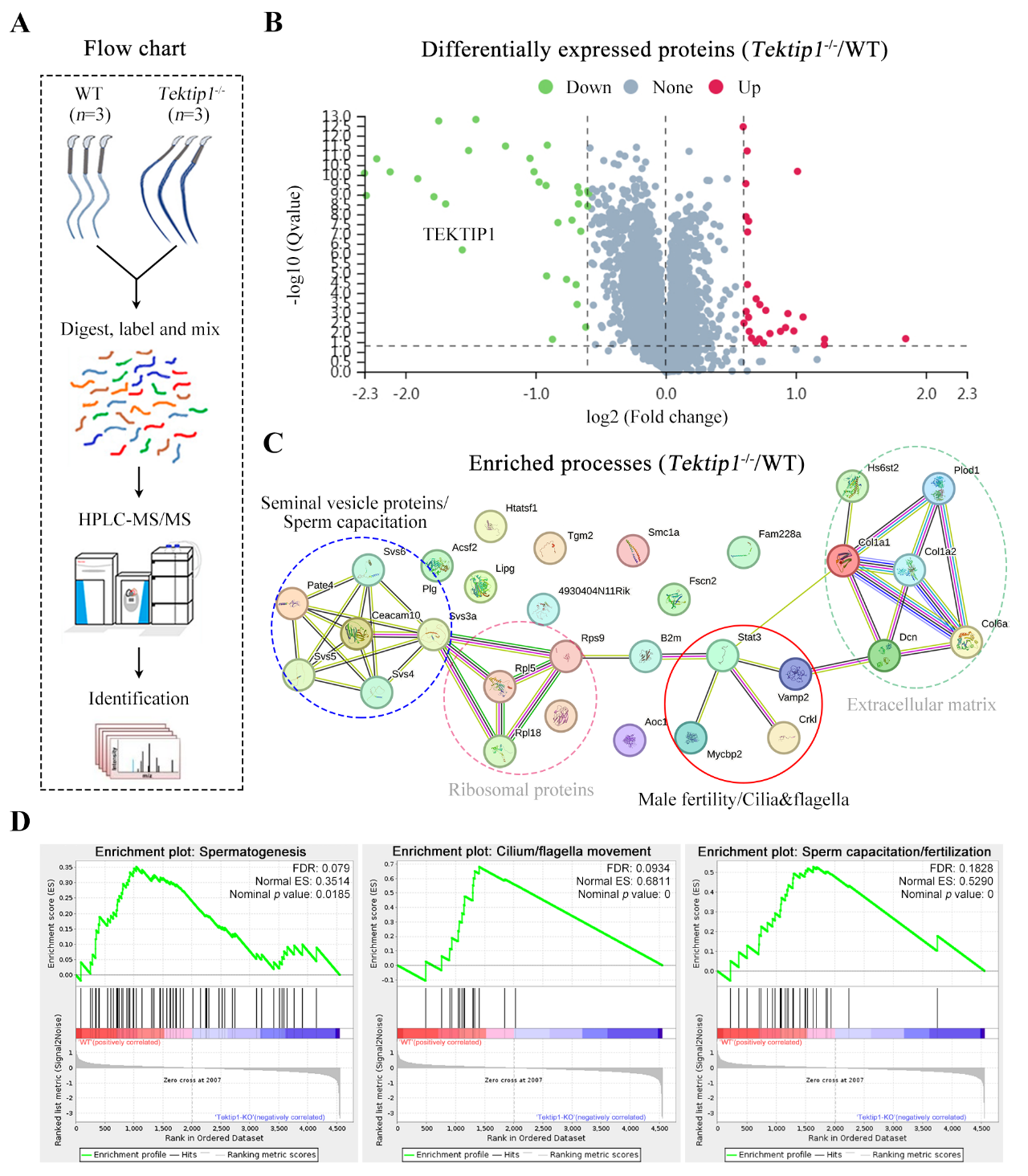


**Fig. S5** **Proteomics analysis of sperm samples from WT mice and *Tektip1*^-/-^ mice. A** Flow chart of proteomics analysis using mouse sperm (*n*=3 each group). **B** Volcano plot showed the differentially expressed (DE) proteins of sperm between WT mice and *Tektip1*^-/-^ mice (threshold: fold-change, >1.5; *p* value, < 0.05). **C** The enriched processes of downregulated proteins with significant difference between two groups. KEGG analysis (https://www.kegg.jp/) was performed and the result was presented as protein-protein interaction networks using STRING (https://string-db.org/). **D** Gene set enrichment analysis (GSEA) (https://www.gsea-msigdb.org/gsea/index.jsp) of all DE proteins between two groups to reveal enriched progresses. |Normal ES|>1, Normal *p* value<0.05, FDR<0.25.

**
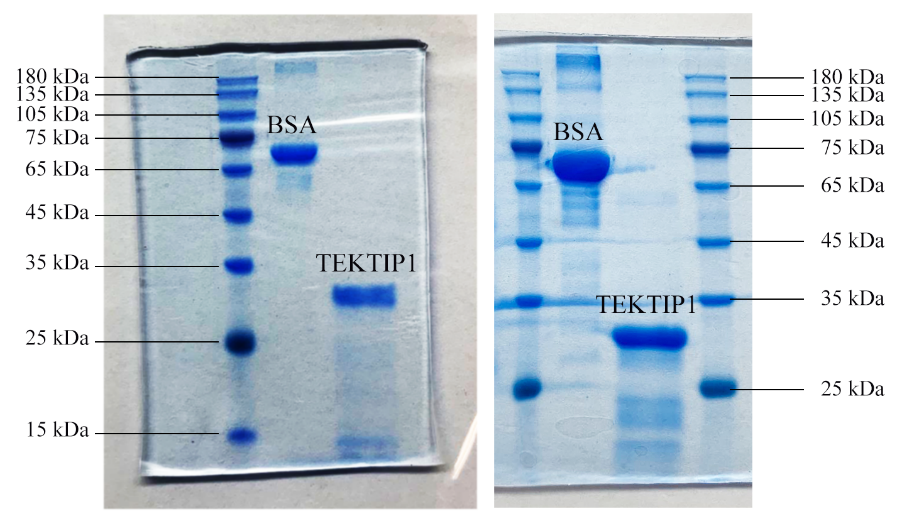
**

**Fig. S6. Coomassie brilliant blue stain of the gel of purified TEKTIP1.** Full-length mouse TEKTIP1 was cloned into the pET-N-His-C-His vector and then transfected into the ER2566 E. coli strain. Protein expression was induced by 1 mM IPTG at 30°C overnight. After centrifugation, the bacterial pellet was resuspended in buffer (50 mM Tris-HCl pH 8.0, 200 mM NaCl), and the proteins were released by sonication. After centrifugation, anti-His beads were added to the supernatant and incubated overnight at 4°C. After washing, recombinant protein was eluted with 250 mM imidazole.

BSA (standard grade) was utilized as control.

**Table S1. Differentially expressed proteins in sperm samples between WT mice and *Tektip1*^-/-^ mice.**

| **Symbol** | **Gene ID** | **NCBI Protein ID** | **Log2FC(KO / WT)** | ***P*-value** |
| --- | --- | --- | --- | --- |
| Hs6st2 | 50786 | NP_001277396 | -2.313892919 | 8.20E-11 |
| Mycbp2 | 105689 | XP_017171268 | -2.299760218 | 1.12E-09 |
| Svs6 | 20945 | NP_038707 | -2.219540144 | 1.52E-11 |
| Pate4 | 56872 | NP_064660 | -2.116486021 | 7.02E-11 |
| Svs3a | 64335 | NP_067338 | -1.904500613 | 1.56E-10 |
| Svs5 | 20944 | NP_033327 | -1.780185495 | 1.29E-09 |
| Vamp2 | 22318 | NP_033523 | -1.743287384 | 1.80E-13 |
| Aoc1 | 76507 | XP_030111522 | -1.689991316 | 2.99E-09 |
| Svs4 | 20941 | NP_033326 | -1.562132944 | 6.52E-07 |
| LOC118567893 | 118567893 | XP_036013017 | -1.513504096 | 5.76E-12 |
| Tektip1 | 432479 | NP_001014836 | -1.457719472 | 1.52E-13 |
| Smc1a | 24061 | NP_062684 | -1.229936645 | 3.41E-12 |
| Plg | 18815 | NP_032903 | -1.041807584 | 1.45E-11 |
| Col1a1 | 12842 | NP_031768 | -1.010817264 | 6.93E-11 |
| B2m | 12010 | NP_033865 | -0.971825306 | 2.31E-10 |
| Col1a2 | 12843 | NP_031769 | -0.91859969 | 3.46E-10 |
| Fam228a | 74855 | NP_083383 | -0.91414809 | 1.38E-05 |
| Ceacam10 | 26366 | NP_031701 | -0.90859364 | 3.08E-12 |
| Hyi | 68180 |  | -0.868979827 | 0.002293568 |
| Fscn2 | 238021 | NP_766390 | -0.827101068 | 2.67E-08 |
| Crkl | 12929 | NP_031790 | -0.760794382 | 2.06E-05 |
| Dcn | 13179 | NP_031859 | -0.71911518 | 1.99E-08 |
| Plod1 | 18822 | NP_035252 | -0.687297088 | 3.83E-05 |
| Stat3 | 20848 | NP_998824 | -0.679814658 | 3.86E-04 |
| Tgm2 | 21817 | NP_033399 | -0.674456381 | 4.05E-10 |
| Rps9 | 76846 | NP_084043 | -0.669493283 | 2.98E-09 |
| Rpl5 | 100503670 | NP_058676 | -0.66202038 | 8.07E-10 |
| Col6a1 | 12833 | NP_034063 | -0.651632097 | 7.35E-08 |
| Acsf2 | 264895 | NP_722502 | -0.613473434 | 0.005511729 |
| Htatsf1 | 72459 | NP_083647 | -0.597635268 | 3.87E-09 |
| Rpl18 | 19899 | NP_033103 | -0.597534385 | 6.55E-10 |
| Lipg | 16891 | NP_034850 | -0.590148393 | 8.65E-10 |
| Hbb-bt | 101488143 | NP_032246 | 0.301694337 | 9.33E-07 |
| Hba-a1 | 15122 | NP_032244 | 0.596494855 | 3.57E-13 |
| Haghl | 68977 | NP_081173 | 0.602423444 | 0.003407235 |
| Diablo | 66593 | NP_075721 | 0.617170534 | 2.78E-10 |
| Cox7a2l | 20463 | NP_033213 | 0.618685547 | 1.33E-08 |
| Tor1a | 30931 | NP_659133 | 0.619695042 | 8.74E-04 |
| Myo10 | 17909 | NP_062345 | 0.629367963 | 3.76E-05 |
| Lyz2 | 17105 | NP_059068 | 0.630369574 | 8.00E-08 |
| Ppp2r3c | 59032 | NP_067504 | 0.637631171 | 0.001753292 |
| Ppp1r2 | 66849 | NP_080076 | 0.638326312 | 2.25E-08 |
| Llgl2 | 217325 | NP_663413 | 0.643125501 | 0.009024336 |
| Cacna1c | 12288 | NP_001153005 | 0.660861439 | 0.01982279 |
| Fras1 | 231470 | NP_780682 | 0.692293476 | 0.03122794 |
| Mterf3 | 66410 | NP_079823 | 0.696121905 | 1.99E-04 |
| Adgrf2 | 435529 | NP_001028665 | 0.720821286 | 0.02216536 |
| Ccdc124 | 234388 | NP_081240 | 0.724767783 | 3.95E-04 |
| Auts2 | 319974 | NP_001350409 | 0.752768466 | 0.03558297 |
| Osgep | 66246 | NP_598437 | 0.770455188 | 7.77E-04 |
| Ankrd31 | 625662 | NP_001357857 | 0.802116727 | 0.01168817 |
| Adam26a | 13525 | NP_034215 | 0.883380555 | 0.009008587 |
| Disc1 | 244667 | XP_011246690 | 0.922555497 | 0.005834775 |
| Arel1 | 68497 | XP_036013493 | 0.940207071 | 0.001129129 |
| Scrib | 105782 | NP_001297472 | 0.985946098 | 0.008653339 |
| Neurod4 | 11923 | NP_031527 | 1.014248478 | 6.62E-11 |
| Fam160b1 | 226252 | NP_663480 | 1.0567883 | 0.001693 |
| 1700025G04Rik | 69399 | XP_011246371 | 1.22001667 | 0.04308023 |
| Nrde2 | 217827 | NP_001277232 | 1.221936776 | 0.02228044 |
| Lamc1 | 226519 | NP_034813 | 1.845485715 | 0.02140766 |

**Table S2. Expression of tektins and microtubule inner proteins (MIPs) in sperm lysates between WT mice and *Tektip1*^-/-^ mice.**

| **Symbol** | **Gene ID** | **NCBI Protein ID** | **Log2FC(KO / WT)** | ***P*-value(KO / WT)** |
| --- | --- | --- | --- | --- |
| Tekt1 | 21689 | NP_001268936 | 0.062811633 | 1.37E-05 |
| Tekt2 | 24084 | NP_001344191 | 0.051240077 | 4.36E-05 |
| Tekt3 | 71062 | NP_081936 | 0.052894811 | 1.28E-06 |
| Tekt4 | 71840 | NP_082227 | 0.03900402 | 7.01E-06 |
| Tekt5 | 70426 | NP_001277930 | 0.041743168 | 3.03E-07 |
| 4930404N11Rik | 432479 | NP_001014836 | -1.457719472 | 1.52E-13 |
| 1700013F07Rik | 75504 | NP_083590 | -0.078564619 | 0.002492803 |
| Cfap20 | 14894 | NP_032213 | -0.059477048 | 0.07817659 |
| Pacrg | 69310 | NP_081308 | -0.035612814 | 0.01547537 |
| Tex37 | 74221 | NP_083101 | -0.015324987 | 0.4580874 |
| 1110032A03Rik | 68721 | NP_075972 | -0.009589714 | 0.4370535 |
| Fam166a | 68222 | NP_080900 | 6.97E-04 | 0.8725487 |
| Tepp | 73407 | NP_955534 | 0.008733115 | 0.5843011 |
| Ppp1r32 | 67752 | XP_017173771 | 0.008789379 | 0.1140757 |
| Fam183b | 75429 | NP_083559 | 0.01194477 | 0.382115 |
| 1700007K13Rik | 69327 | NP_081316 | 0.015413669 | 0.5195484 |
| 1700001L19Rik | 69315 | NP_081311 | 0.020632503 | 0.13847 |
| 1700012P22Rik | 69364 | NP_081332 | 0.026695192 | 0.135273 |
| 1110017D15Rik | 73721 | NP_001041470 | 0.029422972 | 0.01724332 |
| Fam166c | 75434 | NP_083561 | 0.030169598 | 0.01072166 |
| Cfap53 | 74453 | NP_083224 | 0.031219179 | 0.01301438 |
| Enkur | 71233 | NP_082004 | 0.033305897 | 0.1563786 |
| Efhc2 | 74405 | NP_083192 | 0.036686774 | 4.71E-04 |
| Cfap45 | 71870 | NP_082248 | 0.0418675 | 2.73E-04 |
| Spag8 | 433700 | NP_001277391 | 0.047272102 | 0.0120694 |
| Efhb | 211482 | NP_766085 | 0.049358689 | 1.39E-06 |
| Tex43 | 67343 | NP_080375 | 0.050516795 | 0.145915 |
| Efcab6 | 77627 | XP_036015520 | 0.053653231 | 3.67E-05 |
| Cfap126 | 75472 | NP_001366356 | 0.056880594 | 0.002478469 |
| Efhc1 | 71877 | NP_082250 | 0.057449689 | 9.43E-07 |
| Mns1 | 17427 | NP_032639 | 0.059522856 | 6.09E-04 |
| 1700028P14Rik | 67483 | NP_080464 | 0.062961484 | 1.59E-06 |
| Ribc2 | 67747 | NP_080633 | 0.068319165 | 1.22E-08 |
| Spaca9 | 69987 | NP_081559 | 0.070696702 | 0.001455207 |
| Cfap161 | 75556 | NP_083611 | 0.074932137 | 2.71E-05 |
| Ribc1 | 66611 | NP_079936 | 0.075884123 | 2.94E-05 |
| Nme7 | 171567 | NP_001280242 | 0.077598105 | 6.66E-05 |
| Ccdc173 | 75051 | NP_001071152 | 0.077789463 | 2.82E-04 |
| Dusp21 | 73547 | NP_082844 | 0.08199288 | 0.005447134 |
| Ccdc105 | 70976 | NP_081906 | 0.088769049 | 1.39E-04 |
| 1700094D03Rik | 73545 | NP_082843 | 0.089184344 | 3.99E-06 |
| Cfap52 | 71860 | NP_082239 | 0.10691326 | 2.29E-04 |

**Table S3. Primers for plasmid construction.**

| **Gene** | **Primer** | **Sequence (5’→3’)** | **Size** |
| --- | --- | --- | --- |
| *TEKT1* | F | GGGGTACCCCATGGCCAAGCTACTGCAATCTCC | 1257 bp |
|  | R | TGCTCTAGAGCAGCAGATGGCCTCTGCGCG |  |
| *TEKT2* | F | GGGGTACCCCATGGCGACACTAAGTTTCAAGC | 1293 bp |
|  | R | TGCTCTAGAGCAGGTTAGCTCCAGCTGGCAG |  |
| *TEKT3* | F | ATAAGAATGCGGCCGCTAAACATAATGGAACTTTTAGGTTCTACTTTGACG | 1473 bp |
|  | R | TGCTCTAGAGCAGCAGTAGCCCACCAGCCG |  |
| *TEKT4* | F | GGGGTACCCCATGGCCCAAACGGGCGT | 1344 bp |
|  | R | TGCTCTAGAGCACTGGTAGCCAGCCAGCTGGA |  |
| *TEKTIP1* | F | CCCAAGCTTGGGATGGAAAACGTGAGGCGGGAG | 618 bp |
|  | R | CCGCTCGAGCGGGAGCGCGGGTGTGAAAGCC |  |

Endonuclease sites and protective bases were underlined.
